# ROBIS: A new tool to assess risk of bias in systematic reviews was developed

**DOI:** 10.1016/j.jclinepi.2015.06.005

**Published:** 2016-01

**Authors:** Penny Whiting, Jelena Savović, Julian P.T. Higgins, Deborah M. Caldwell, Barnaby C. Reeves, Beverley Shea, Philippa Davies, Jos Kleijnen, Rachel Churchill, ROBIS group

**Affiliations:** a School of Social and Community Medicine, University of Bristol, Canynge Hall, 39 Whatley Road, Bristol BS8 2PS, UK; b The National Institute for Health Research Collaboration for Leadership in Applied Health Research and Care West at University Hospitals Bristol NHS Foundation Trust, 9th Floor, Whitefriars, Lewins Mead, Bristol BS1 2NT; c Kleijnen Systematic Reviews Ltd, Unit 6, Escrick Business Park, Riccall Road, Escrick, York YO19 6FD, UK; d Centre for Reviews and Dissemination, University of York, York YO10 5DD, UK; e School of Clinical Sciences, University of Bristol, Bristol Royal Infirmary, Level Queen's Building, 69 St Michael's Hill, Bristol BS2 8DZ, UK; f Community Information and Epidemiological Technologies Institute of Population Health, 1 Stewart Street, Room 319, Ottawa, Ontario, K1N 6N5, Canada; g School for Public Health and Primary Care (CAPHRI), Maastricht University, PO Box 616, 6200 MD, Maastricht, The Netherlands

**Keywords:** Evidence, Meta-analysis, Quality, Risk of bias, Systematic review, Tool

## Abstract

**Objective:**

To develop ROBIS, a new tool for assessing the risk of bias in systematic reviews (rather than in primary studies).

**Study Design and Setting:**

We used four-stage approach to develop ROBIS: define the scope, review the evidence base, hold a face-to-face meeting, and refine the tool through piloting.

**Results:**

ROBIS is currently aimed at four broad categories of reviews mainly within health care settings: interventions, diagnosis, prognosis, and etiology. The target audience of ROBIS is primarily guideline developers, authors of overviews of systematic reviews (“reviews of reviews”), and review authors who might want to assess or avoid risk of bias in their reviews. The tool is completed in three phases: (1) assess relevance (optional), (2) identify concerns with the review process, and (3) judge risk of bias. Phase 2 covers four domains through which bias may be introduced into a systematic review: study eligibility criteria; identification and selection of studies; data collection and study appraisal; and synthesis and findings. Phase 3 assesses the overall risk of bias in the interpretation of review findings and whether this considered limitations identified in any of the phase 2 domains. Signaling questions are included to help judge concerns with the review process (phase 2) and the overall risk of bias in the review (phase 3); these questions flag aspects of review design related to the potential for bias and aim to help assessors judge risk of bias in the review process, results, and conclusions.

**Conclusions:**

ROBIS is the first rigorously developed tool designed specifically to assess the risk of bias in systematic reviews.

**What is new?**

This article describes ROBIS, a new tool for assessing the risk of bias in systematic reviews (rather than in primary studies)

**Key findings:**
• ROBIS has been developed using rigorous methodology and is currently aimed at four broad categories of reviews mainly within healthcare settings: interventions, diagnosis, prognosis and aetiology.
• The tool is completed in 3 phases: (1) assess relevance (optional), (2) identify concerns with the review process and (3) judge risk of bias.
• Phase 2 covers four domains through which bias may be introduced into a systematic review: study eligibility criteria; identification and selection of studies; data collection and study appraisal; and synthesis and findings.
• Phase 3 assesses the overall risk of bias in the interpretation of review findings and whether this considered limitations identified in any of the Phase 2 domains.

**What this adds to what was known?**
• Systematic reviews are generally considered to provide the most reliable form of evidence to guide decision makers. Systematic flaws or limitations in the design or conduct of a review have the potential to bias results. Several tools exist for undertaking critical appraisal and quality assessment of systematic reviews but none specifically aim to assess the risk of bias in systematic reviews. We developed the ROBIS tool to fill this gap in risk of bias assessment tools.

**What is the implication and what should change now?**
• We hope that ROBIS will help improve the process of risk of bias assessment in overviews and guidelines, leading to more robust recommendations for improvements in patient care.

## 1 Introduction

Systematic reviews are generally considered to provide the most reliable form of evidence for the effects of a medical intervention, test, or marker [1], [2]. They can be used to address questions on a wide range of topics using studies of varying designs. Increasingly, standards for evidence-based guidelines stipulate the use of systematic reviews [3], which can in turn determine care pathways and coverage for services, therapies, drugs, and so on. Because systematic reviews serve a vital role in clinical decision making and resource allocation, decision makers should expect consistent and unbiased standards across topics.

Systematic flaws or limitations in the design or conduct of a review have the potential to bias results. Bias can arise at all stages of the review process; users need to consider these potential biases when interpreting the results and conclusions of a review. The potential of flaws in the design and conduct of systematic reviews is becoming better understood. Producers of systematic reviews focus increasingly on preventing potential biases in their reviews by developing explicit expectations for their conduct. For example, the Cochrane Collaboration has formally adopted the MECIR (Methodological Expectations of Cochrane Intervention Review) guidelines [4], and the US Institute of Medicine has recommended standards for conducting high-quality systematic reviews [5]. The development and adoption of the PRISMA statement [6], [7] has led to improvements in reporting of systematic reviews. This helps readers to assess whether appropriate steps have been taken to minimize bias in the design and conduct of the review.

Several tools exist for undertaking critical appraisal and quality assessment of systematic reviews. Although none has become universally accepted, the AMSTAR tool is probably the most commonly used quality assessment tool for systematic reviews [8]. We are not aware of any tool designed specifically to assess the risk of bias in systematic reviews; all currently available tools have a broader objective of critical appraisal [8], [9] or focus specifically on meta-analyses [10]. We developed the ROBIS tool, described in the following, to fill this gap in risk of bias assessment tools.

## 2 Development of ROBIS

Development of ROBIS was based on a four-stage approach [11]: define the scope, review the evidence base, hold a face-to-face meeting, and refine the tool through piloting.

### 2.1 Development stage 1—define the scope

We established a steering group of 11 experts in the area of systematic reviews. This group agreed on key features of the desired scope of ROBIS through regular teleconferences. The scope was further refined during the face-to-face meeting and during a Delphi procedure which was also used to finalize tool content. We agreed that ROBIS would assess both the risk of bias in a review and (where appropriate) the relevance of a review to the research question at hand. Specifically, it addresses (1) the extent to which the research question addressed by the review matches the research question being addressed by its user (eg, an overview author or guideline developer) and (2) the degree to which the review methods minimized the risk of bias in the summary estimates and review conclusions. Evidence from a review may have “limited relevance” if the review question does not match the overview or guideline question. “Bias” occurs if systematic flaws or limitations in the design, conduct, or analysis of a review distort the review results or conclusions. The distinction between bias in the review (sometimes called “metabias”) and bias in the primary studies included in the review is important. A systematic review could be classed as low risk of bias even if the primary studies included in the review are all at high risk of bias, as long as the review has appropriately assessed and considered the risk of bias in the primary studies when drawing the review conclusions.

A key aim of the development of the ROBIS tool was that the tool structure be as generic as possible but initially focus on systematic reviews covering questions relating to effectiveness (interventions), etiology, diagnosis, and prognosis (see Box for examples of each review type). ROBIS should be usable by reviewers with different backgrounds, although it was accepted that some methodologic and/or content expertise would be required. The tool needed to be able to distinguish between reviews at high and low risk of bias. We defined the target audience of ROBIS as guideline developers, authors of overviews of systematic reviews (“reviews of reviews”), and review authors who might want to assess or avoid risk of bias in their reviews.

**Box:** Examples of target questions and PICO equivalents for different types of systematic review

Review type	PICO equivalent	Example
Intervention [12]	Patients/population(s)	Adults with chronic hepatitis C virus infection
Intervention(s)	Triple antiviral therapy with pegylated interferon
Comparator(s)	Dual antiviral therapy
Outcome(s)	Sustained virologic response

Etiology [13]	Patients/population(s)	Adults
Exposure(s) and comparator(s)	Body mass index
Outcome(s)	Colorectal cancer

Diagnosis [14]	Patient(s)	Adults with symptoms suggestive of rectal cancer
Index test(s)	Endoscopic ultrasound
Reference standard	Surgical histology
Target condition	T0 stage of rectal cancer

Prognosis [15]	Patients	Pregnant women, with or without fetal growth restriction, no evidence of premature rupture of membranes, no evidence of congenital or structural anomalies
Outcome to be predicted	Adverse pregnancy outcome (low or high birth weight, neonatal death, perinatal mortality)
Intended use of model	To predicting the effect of ultrasound measurements of amniotic fluid on pregnancy outcome
Intended moment in time	Late pregnancy (>37-wk gestation)

We agreed to adopt a domain-based structure similar to that used in tools designed to assess risk of bias in primary studies (eg, the Cochrane Risk of Bias tool [16], QUADAS-2 [17], ACROBAT-NRS [18], and PROBAST [19]). We also agreed on a three-stage approach to assessing risk of bias/concerns regarding the review process: information used to support the judgment of risk of bias, signaling questions, and judgment. We decided that signaling questions should be answered as “yes,” “probably yes,” “probably no,” “no,” “no information.” Risk of bias is judged as “low,” “high,” or “unclear.”

### 2.2 Development stage 2—review of the evidence base

We used three different approaches to obtain evidence to inform the development of ROBIS. First, we classified the 80 MECIR conduct items [4] as relating to bias, variability/applicability, the reporting quality, or as being a “process” item (ie, items relating to how the review should be conducted from a practical perspective). For each of the 46 items classified as bias items, we developed a suggested “signaling question.” Second, we reviewed 40 existing tools designed to assess the quality of systematic reviews or meta-analyses. We classified items included in the tools according to five areas of bias affecting systematic reviews (question/inclusion criteria, search, review process, synthesis, and conclusions) or as being unrelated to bias. We also recorded details on tool development, tool structure, and interrater reliability and considered how experience and learning from existing tools might inform the development of ROBIS. Third, we conducted a review of overviews that had used the AMSTAR tool to assess the quality of systematic reviews. The aim of this review was to provide information on the requirements of potential users of ROBIS. Full details of the two reviews (on existing tools and overviews using the AMSTAR tool) will be reported separately. On the basis of the evidence accumulated from these three sources, we summarized information on the requirements of ROBIS and identified possible signaling questions for inclusion in the tool.

### 2.3 Development stage 3—face-to-face meeting of the ROBIS group

We held a 1-day face-to-face meeting on 17 September 2013 before the Cochrane Colloquium in Quebec City, Canada. The main objective of this meeting was to develop a first draft of ROBIS. The 21 attendees (including seven steering group members) were invited to provide input from a wide range of user stakeholders, including methodologic experts, experienced systematic reviewers, and guideline developers from different countries and different organizations. Ten invitees who were not able to attend were given the opportunity to contribute to the development of ROBIS through involvement in the Delphi process. We outlined the scope of the work and presented summaries of the evidence obtained through stage 2. Initial group discussions focused on whether ROBIS should be restricted to reviews of randomised clinical trials or be more generic in design, the definition of “bias” from the perspective of ROBIS, how we could achieve an overall risk of bias rating for a single review without using summary quality scores, whether conflict of interest/source of funding should be included in ROBIS, and the domains that the tool should cover. This was followed by small group discussions of four to five participants to review the signaling questions within each domain. On the basis of the outputs of this meeting and related feedback, the project leads produced a first draft of the ROBIS tool.

### 2.4 Development stage 4—pilot and refine

We used a modified Delphi process to finalize the scope and content of ROBIS. Online questionnaires were developed to gather structured feedback for each round. Participants in the Delphi process included members of the steering group who had not contributed directly to the development of that round of the survey, participants from the face-to-face meeting, and five additional methodologic experts who were not able to attend the face-to-face meeting. This resulted in a maximum number of respondents of 29 per round. After three rounds of the Delphi, we judged there to be sufficient agreement that no further rounds were necessary. Participants were sent the agreed draft version of ROBIS and given the opportunity to provide any final comments. A final draft version of the tool was then produced to be evaluated in the piloting stage of the project.

We held three workshops on ROBIS: two for systematic reviewers in York, UK (May and October 2014) and one at the Cochrane Colloquium in Hyderabad (September 2014). These gave participants the opportunity to pilot the tool and provide feedback on the practical issues associated with using the tool, which were then incorporated into the guidance document. Three independent pairs of reviewers working on overviews piloted a draft version of the tool and provided structured feedback. Information on interrater agreement was available for one pair of reviewers, who were independent of the tool developers, assessing eight reviews; four Cochrane reviews, and four non-Cochrane reviews. This gave information on 40 domain-rating pairs (five ROBIS domains assessed for eight reviews by two reviewers). The reviewers agreed on the ratings for 26 (65%) domains and disagreed for 14. Most disagreements arose from one reviewer assigning a rating of unclear and the other assigning a rating of high or low. There were only four domains (10%) for which one reviewer assigned a rating of high and the other assigned a rating of low. The reviewers agreed on all domain-level ratings for three of the eight reviews; these reviews were rated as low for all domains. The synthesis and findings domain caused the most disagreements in ratings. Three reviews were rated as low by both reviewers for this domain, there was disagreement in how to rate the other five reviews. All other domains were rated as low by both reviewers for at least five of the eight reviews. Agreement was greater for Cochrane reviews than for non-Cochrane reviews: for the 20 domain-rating pairs for Cochrane reviews, there were only two domains where the reviewers disagreed. There are two possible explanations for this. The first is that it appears that there was better agreement for reviews judged to be at low risk of bias. The Cochrane reviews generally rated better on the ROBIS assessment, and so, it may be that they were easier to assess using ROBIS because they were at lower risk of bias. A second possible explanation is that Cochrane reviews usually contain more detailed information on review methods which may make it easier to apply ROBIS. This could suggest that difficulties in applying ROBIS are more down to limitations in reporting of reviews rather than to difficulties in applying the tool itself. This was supported by examining the “support for judgment” statements extracted by the two reviewers and the answers to the individual signaling questions which suggested that some of the discrepancies in domain-level ratings were the result of differences in how lack of reported information was handled by the two reviewers.

On the basis of the results of the piloting, we produced a final version of ROBIS and an accompanying guidance document.

## 3 The ROBIS tool

The full ROBIS tool and guidance documents are available from the ROBIS Web site (www.robis-tool.info) and as Appendices at www.jclinepi.com.

The tool is completed in three phases: (1) assess relevance (optional), (2) identify concerns with the review process, and (3) judge risk of bias in the review. Signaling questions are included to help assess specific concerns about potential biases with the review. The ratings from these signaling questions help assessors to judge overall risk of bias. Table 1 summarizes the phase 2 domains, phase 3, and signaling questions within each domain. A detailed overview of each domain and guidance on how to rate each signaling question are provided in the guidance document.

### 3.1 ROBIS phase 1: assessing relevance (optional)

Assessors first report the question that they are trying to answer (eg, in their overview or guideline)—we have called this the “target question.” For effectiveness reviews, they are asked to define this in terms of the PICO (participants, interventions, comparisons, and outcomes). For reviews of different types of questions (eg, diagnostic test, prognostic factors, etiology, or prediction models), alternative categories are provided as appropriate (see Box). Assessors complete the PICO or equivalent for the systematic review to be assessed using ROBIS and are then asked whether the two questions (target question and systematic review question) match. If one or more of the categories (PICO or equivalent) do not match, then this should be rated as “no.” If there is a partial match between categories, then this should be rated as “partial.” For example, if the target question relates to adults, the systematic review is restricted to participants aged >60 years. If a review is being assessed in isolation and there is no target question, then this phase of ROBIS can be omitted.

### 3.2 ROBIS phase 2: identifying concerns about bias in the review process

Phase 2 aims to identify areas where bias may be introduced into the systematic review. It involves the assessment of four domains to cover key review processes: study eligibility criteria; identification and selection of studies; data collection and study appraisal; and synthesis and findings. This phase of ROBIS identifies areas of potential concern to help judge overall risk of bias in the final phase. Each domain comprises three sections: information used to support the judgment, signaling questions, and judgment of concern about risk of bias. The domains should be considered sequentially and not assessed as stand-alone units. For example, this means that, when assessing domain 2 (identification and selection of studies), the assessor should consider the searches in relation to the research question specified in domain 1.

The signaling questions are answered as “yes,” “probably yes,” “probably no,” “no,” and “no information,” with “yes” indicating low concerns. The subsequent level of concern about bias associated with each domain is then judged as “low,” “high,” or “unclear.” If the answers to all signaling questions for a domain are “yes” or “probably yes,” then level of concern can be judged as low. If any signaling question is answered “no” or “probably no,” potential for concern about bias exists. The “no information” category should be used only when insufficient data are reported to permit a judgment. By recording the information used to reach the judgment (“support for judgment”), we aim to make the rating transparent and, where necessary, facilitate discussion among review authors completing assessments independently. ROBIS users are likely to need both subject content and methodologic expertise to complete an assessment.

#### 3.2.1 Domain 1: study eligibility criteria

The first domain aims to assess whether primary study eligibility criteria were prespecified, clear, and appropriate to the review question. A systematic review should begin with a clearly focused question or objective [1]. This should be reflected in the prespecification of criteria used for deciding whether primary studies are eligible for inclusion in the review. This prespecification aims to ensure that decisions about which studies to include are made consistently rather than on existing knowledge about the characteristics and findings of the studies themselves. It is usually only possible to assess whether eligibility criteria have been appropriately prespecified (and adhered to in the review) if a protocol or registration document is available which predates the conduct and reporting of the review. When no such document is available, assessors will need to base their judgment about this domain on the report of the review findings, making it difficult to know whether these criteria were actually stipulated in advance and governed what the reviewers did throughout the review, or whether they were decided or modified during the review process.

#### 3.2.2 Domain 2: identification and selection of studies

This domain aims to assess whether any primary studies that would have met the inclusion criteria were not included in the review. A sensitive search to retrieve as many eligible studies as possible is a key component of any systematic review. Ideally, this search is carried out by or with guidance from a trained information specialist. Unbiased selection of studies based on the search results helps to ensure that all relevant studies identified by the searches are included in the review. Searches should involve appropriate databases and electronic sources (which index journals, conferences, and trial records) to identify published and unpublished reports, include methods additional to database searching to identify reports of eligible studies (eg, checking references in existing reviews, citation searching, hand-searching) and use of an appropriate and sensitive search strategy. Search strategies should include free-text terms (eg, in the title and abstract) and any suitable subject indexing (eg, MeSH or EMTREE) likely to identify relevant studies. It can be difficult to assess the sensitivity of a search strategy without methodologic knowledge relating to searching practice and content expertise relating to the review topic. In general, assessors should consider whether an appropriate range of terms is included to cover all possible ways in which the concepts used to capture the research question could be described.

The process of selecting studies for inclusion in the review once the search results have been compiled is also covered by this domain. This involves screening titles and abstracts and assessing full-text studies for inclusion. To minimize the potential for bias and errors in these processes, titles and abstracts should be screened independently by at least two reviewers and full-text inclusion assessment should involve at least two reviewers (either independently or with one performing the assessment and the second checking the decision).

#### 3.2.3 Domain 3: data collection and study appraisal

The third domain aims to assess whether bias may have been introduced through the data collection or risk of bias assessment processes. Rigorous data collection should involve planning ahead at the protocol stage and using a structured data collection form that has been piloted. All data that will contribute to the synthesis and interpretation of results should be collected. These data should include both numerical and statistical data and more general primary study characteristics. If data are not available in the appropriate format required to contribute to the synthesis, review authors should report how these data were obtained. For example, primary study authors may be contacted for additional data. Appropriate statistical transformations may be used to derive the required data. Data extraction creates the potential for error. Errors could arise from mistakes when transcribing data or failing to collect relevant information that is available in a study report. Bias may also arise from the process of data extraction which is, by its nature, subjective and open to interpretation. Duplicate data extraction (or single data extraction with rigorous checking) is therefore essential to safeguard against random errors and potential bias [2].

Validity of included studies should be assessed using appropriate criteria given the design of the primary studies included in the review [1], [2]. This assessment may be carried out using a validated tool developed specifically for studies of the design being evaluated or may simply be a list of relevant criteria that may be important potential sources of bias. Whether a published tool or ad hoc criteria are used, the assessor should consider whether the criteria are sufficient to identify all important potential sources of bias in the included studies. As with data extraction, bias or error can occur in the process of risk of bias assessment. Risk of bias assessment should, therefore, involve two reviewers, ideally working independently but at a minimum, the second reviewer checking the decisions of the first reviewer.

#### 3.2.4 Domain 4: synthesis and findings

This domain aims to assess whether, given a decision has been made to combine data from the included primary studies (either in a quantitative or nonquantitative synthesis), the reviewers have used appropriate methods to do so. Approaches to synthesis depend on the nature of the review question being addressed and on the nature of the primary studies being synthesized. For randomised clinical trials, a common approach is to take a weighted average of treatment effect estimates (on the logarithmic scale for ratio measures of treatment effect), weighting by the precisions of the estimates [20]. Either fixed-effect or random-effect models can be assumed for this. However, there are many variants and extensions to this, with the options of modeling outcome data explicitly (for example, taking a logistic regression approach for binary data [21], of modeling two or more outcomes simultaneously (bivariate or multivariate meta-analysis [22], of modeling multiple treatment effects simultaneously (network meta-analysis [23]), or of modeling variation in treatment effects (metaregression [24]), and these can be combined, making the synthesis very complex. Similar options are available for other types of review questions. For diagnostic test accuracy, a bivariate approach has become standard, in which sensitivity and specificity are modeled simultaneously to take account of their correlation [25]. For some reviews, a statistical synthesis may not be appropriate and instead a nonquantitative or narrative overview of results should be reported.

Some of the most important aspects to consider in any synthesis (either quantitative or nonquantitative) are (1) whether the analytic approach is appropriate for the research question posed; (2) whether between-study variation (heterogeneity) is taken into account; (3) whether biases in the primary studies are taken into account; (4) whether the information from the primary studies being synthesized is complete (particularly if there is a risk that missing data are systematically different from available data, for example, because of publication or reporting bias); and (5) whether the reviewers have introduced bias in the way that they report their findings. Technical aspects of the meta-analysis method, such as the choice of estimation method, are unlikely to be an important consideration. However, mistakes may be important, such as interpreting standard errors as standard deviations, failing to adjust for design issues such as matched or clustered data, or applying the standard weighted-average approach to risk ratios rather than their logarithms.

### 3.3 ROBIS phase 3: judging risk of bias

The final phase considers whether the systematic review as a whole is at risk of bias. This assessment uses the same structure as the separate phase 2 domains, including signaling questions and information used to support the judgment, but the judgment regarding concerns about bias is replaced with an overall judgment of risk of bias. The first signaling question for this phase asks whether the interpretation of findings addresses all the concerns identified in domains 1 to 4. If no concerns were identified, then this can be rated as “yes.” If one or more concerns were identified for any of the previous domains but these were appropriately considered when interpreting results and drawing conclusions, then this may also be rated as “yes,” and depending on the rating of the other signaling questions, the review may still be rated as “low risk of bias.” This phase also includes a further three signaling questions relating to the interpretation of the review findings.

### 3.4 Presenting ROBIS assessments

**Fig. 1:**
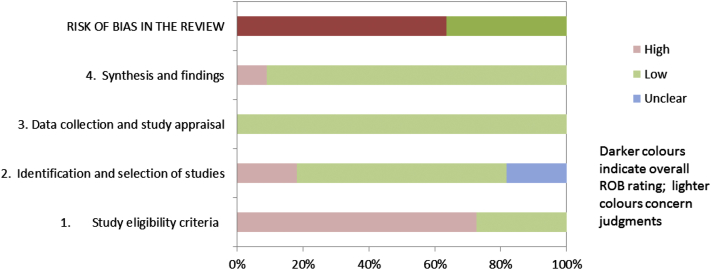
Suggested graphical presentation for ROBIS results from multiple reviews.

**Fig. 2:**
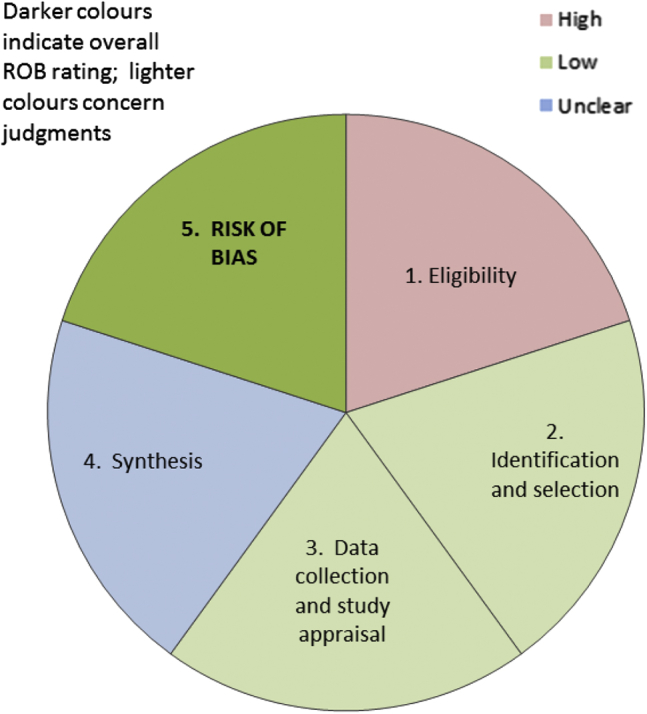
Suggested graphical presentation for ROBIS results from single review: each colored segment shows the concerns for one of the phase 2 ROBIS domains; the final segment (shaded darker) shows the phase risk of bias assessment.

At a minimum, overviews and guidelines should summarize the results of the ROBIS assessment for all included systematic reviews. This could include summarizing the number of systematic reviews that had a low, high, or unclear concern for each phase 2 domain and the number of reviews at high or low risk of bias. Where used, a summary of the relevance assessment should be provided. Reviewers may choose to highlight particular signaling questions on which systematic reviews consistently rated poorly or well. Tabular (Table 2) and graphic (Fig. 1) displays may be useful for summarizing ROBIS assessments across multiple reviews. When using the graphical display, reviewers may consider it more appropriate to weight this figure on the basis of the number of studies included in the review, or the total number of participants in each review, rather than simply on individual reviews. Alternatively, reviewers or guideline developers may choose to include only the review that is most relevant to their target question and at lowest risk of bias. We have also suggested a graphical display to present the results of a ROBIS assessment (each domain rating and overall rating) for a single review (Fig. 2). We emphasize that ROBIS should not be used to generate a summary “quality score” because of the well-known problems associated with such scores [26], [27].

## 4 Discussion

ROBIS is the first rigorously developed tool designed specifically to assess the risk of bias in systematic reviews. The use of a domain-based approach supported by signaling questions follows the most recent methods for developing risk of bias tools. We hope that ROBIS will help improve the process of risk of bias assessment in overviews and guidelines, leading to more robust recommendations for improvements in patient care.

We feel that the approach adopted for the development of ROBIS has a number of strengths. The smaller steering group enabled us to have initial focused discussions regarding the desired scope of ROBIS. We then involved a wider group through the face-to-face meeting and subsequent Web-based Delphi procedure, building on the existing evidence base summarized from our initial evidence reviews. The wider ROBIS group aimed to include all potential user stakeholders such as methodologic experts, systematic reviewers, and guideline developers from a variety of countries and organizations to ensure that ROBIS would meet the needs of all potential users. To avoid excluding potential stakeholders who could not attend the face-to-face meeting, we invited them to participate in the development process through involvement in the Web-based Delphi survey. Initial piloting of ROBIS involved workshops in York and at the Cochrane Colloquium and through volunteers using the tool in their reviews. A potential limitation of our piloting of the tool is that, to date, this has mainly been done by those undertaking overviews of reviews. We are now working with a range of guideline developers who are using the tool, and we will continue to target these groups in future user-testing activities. Further refinement to the guidance documents will continue, and we invite further comment and feedback via the ROBIS Web site and have developed a Web-based survey for this purpose. We plan to use the soon-to-be-launched LATITUDES Network (www.latitudes-network.org) to gather further feedback and increase awareness of ROBIS. LATITUDES is a new initiative similar to the EQUATOR Network that aims to increase the use of key risk of bias assessment tools, help people to use these tools more effectively, improve incorporation of results of the risk of bias assessment into the review, and to disseminate best practice in risk of bias assessment. We hope that LATITUDES will highlight ROBIS as the key risk of bias tool for the assessment of risk of bias in systematic review.

ROBIS is currently aimed at four broad categories of reviews mainly within health care setting, those covering: interventions, diagnosis, prognosis, and etiology. As we build experience of using ROBIS in these reviews, we will consider whether it is appropriate to expand ROBIS to other types of review, including to areas outside health care, and if so whether any modifications are needed either to the tool itself or to the accompanying guidance documents.

## Figures and Tables

**Table 1:** Summary of phase 2 ROBIS domains, phase 3, and signaling questions

**Table 2:** Suggested tabular presentation for ROBIS results
