# Supplementary material for: ROBIS: A new tool to assess risk of bias in systematic reviews was developed
Source: J Clin Epidemiol. 2016 Jan;69:225–34. doi: 10.1016/j.jclinepi.2015.06.005 (PMC4687950; doi:10.1016/j.jclinepi.2015.06.005)
Supplement: Appendix A [file mmc1.pdf]

ROBIS: Tool to assess risk of bias in systematic reviews

Phase 1: Assessing relevance (Optional)

ROBIS is designed to assess the risk of bias in reviews with questions relating to interventions, aetiology, diagnosis and prognosis. State your overview/guideline question (target question) and the question being addressed in the review being assessed:

Intervention reviews:

Category	Target question (e.g. overview or guideline)	Review being assessed
Patients/Population(s):		
Intervention(s):		
Comparator(s):		
Outcome(s):		

For aetiology reviews:

Category	Target question (e.g. overview or guideline)	Review being assessed
Patients/Population(s):		
Exposure(s) and comparator(s):		
Outcome(s):		

For DTA reviews:

Category	Target question (e.g. overview or guideline)	Review being assessed
Patients):		
Index test(s):		
Reference standard:		
Target condition:		

For prognostic reviews:

Category	Target question (e.g. overview or guideline)	Review being assessed
Patients:		
Outcome to be predicted:		
Intended use of model:		
Intended moment in time:		

Does the question addressed by the review match the target question?	YES/NO/UNCLEAR

Phase 2: Identifying concerns with the review process

**DOMAIN 1: STUDY ELIGIBILITY CRITERIA**	
Describe the study eligibility criteria, any restrictions on eligibility and whether there was evidence that objectives and eligibility criteria were pre-specified:	
1.1 Did the review adhere to pre-defined objectives and eligibility criteria?	Y/PY/PN/N/NI
1.2 Were the eligibility criteria appropriate for the review question?	Y/PY/PN/N/NI
1.3 Were eligibility criteria unambiguous?	Y/PY/PN/N/NI
1.4 Were all restrictions in eligibility criteria based on study characteristics appropriate (e.g. date, sample size, study quality, outcomes measured)?	Y/PY/PN/N/NI
1.5 Were any restrictions in eligibility criteria based on sources of information appropriate (e.g. publication status or format, language, availability of data)?	Y/PY/PN/N/NI
Concerns regarding specification of study eligibility criteria	LOW/HIGH/UNCLEAR
Rationale for concern:	

**DOMAIN 2: IDENTIFICATION AND SELECTION OF STUDIES**	
Describe methods of study identification and selection (e.g. number of reviewers involved):	
2.1 Did the search include an appropriate range of databases/electronic sources for published and unpublished reports?	Y/PY/PN/N/NI
2.2 Were methods additional to database searching used to identify relevant reports?	Y/PY/PN/N/NI
2.3 Were the terms and structure of the search strategy likely to retrieve as many eligible studies as possible?	Y/PY/PN/N/NI
2.4 Were restrictions based on date, publication format, or language appropriate?	Y/PY/PN/N/NI
2.5 Were efforts made to minimise error in selection of studies?	Y/PY/PN/N/NI
Concerns regarding methods used to identify and/or select studies	LOW/HIGH/UNCLEAR
Rationale for concern:	

**DOMAIN 3: DATA COLLECTION AND STUDY APPRAISAL**	
Describe methods of data collection, what data were extracted from studies or collected through other means, how risk of bias was assessed (e.g. number of reviewers involved) and the tool used to assess risk of bias:	
3.1 Were efforts made to minimise error in data collection?	Y/PY/PN/N/NI
3.2 Were sufficient study characteristics available for both review authors and readers to be able to interpret the results?	Y/PY/PN/N/NI
3.3 Were all relevant study results collected for use in the synthesis?	Y/PY/PN/N/NI
3.4 Was risk of bias (or methodological quality) formally assessed using appropriate criteria?	Y/PY/PN/N/NI
3.5 Were efforts made to minimise error in risk of bias assessment?	Y/PY/PN/N/NI
Concerns regarding methods used to collect data and appraise studies	LOW/HIGH/UNCLEAR
Rationale for concern:	

DOMAIN 4: SYNTHESIS AND FINDINGS	
Describe synthesis methods:	
4.1 Did the synthesis include all studies that it should?	Y/PY/PN/N/NI
4.2 Were all pre-defined analyses reported or departures explained?	Y/PY/PN/N/NI
4.3 Was the synthesis appropriate given the nature and similarity in the research questions, study designs and outcomes across included studies?	Y/PY/PN/N/NI
4.4 Was between-study variation (heterogeneity) minimal or addressed in the synthesis?	Y/PY/PN/N/NI
4.5 Were the findings robust, e.g. as demonstrated through funnel plot or sensitivity analyses?	Y/PY/PN/N/NI
4.6 Were biases in primary studies minimal or addressed in the synthesis?	Y/PY/PN/N/NI
Concerns regarding the synthesis and findings	LOW/HIGH/UNCLEAR
Rationale for concern:	

Y=YES, PY=PROBABLY YES, PN=PROBABLY NO, N=NO, NI=NO INFORMATION

Phase 3: Judging risk of bias

Summarize the concerns identified during the Phase 2 assessment:

Domain	Concern	Rationale for concern
1. Concerns regarding specification of study eligibility criteria		
2. Concerns regarding methods used to identify and/or select studies		
3. Concerns regarding methods used to collect data and appraise studies		
4. Concerns regarding the synthesis and findings		

RISK OF BIAS IN THE REVIEW	
Describe whether conclusions were supported by the evidence:	
A. Did the interpretation of findings address all of the concerns identified in Domains 1 to 4?	Y/PY/PN/N/NI
B. Was the relevance of identified studies to the review's research question appropriately considered?	Y/PY/PN/N/NI
C. Did the reviewers avoid emphasizing results on the basis of their statistical significance?	Y/PY/PN/N/NI
Risk of bias in the review	RISK: LOW/HIGH/UNCLEAR
Rationale for risk:	

Y=YES, PY=PROBABLY YES, PN=PROBABLY NO, N=NO, NI=NO INFORMATION
