# Supplementary material for: ROBIS: A new tool to assess risk of bias in systematic reviews was developed
Source: J Clin Epidemiol. 2016 Jan;69:225–34. doi: 10.1016/j.jclinepi.2015.06.005 (PMC4687950; doi:10.1016/j.jclinepi.2015.06.005)
Supplement: Appendix B [file mmc2.doc]

# ROBIS: Tool to assess risk of bias in systematic reviews

# Guidance on how to use ROBIS

Penny Whiting (Kleijnen Systematic Reviews Ltd, University of Bristol),

Jelena Savović (University of Bristol)

Julian Higgins (University of Bristol),

Deborah Caldwell (University of Bristol)

Barnaby Reeves (University of Bristol),

Beverley Shea (University of Ottawa)

Philippa Davies (University of Bristol),

Jos Kleijnen (Kleijnen Systematic Reviews Ltd, Maastricht University),

Rachel Churchill (University of Bristol)

# Contents

[**The ROBIS Tool** 4](#__RefHeading___Toc401065143)

[Definition of risk of bias 4](#__RefHeading___Toc401065144)

[Target audience 4](#__RefHeading___Toc401065145)

[**Phase 1: Assessing Relevance (optional)** 4](#__RefHeading___Toc401065146)

[Example Relevance Assessment^3^ 6](#__RefHeading___Toc401065147)

[**Phase 2: Identifying concerns with the review process** 7](#__RefHeading___Toc401065148)

[Domain 1: Study eligibility criteria 8](#__RefHeading___Toc401065149)

[Background 8](#__RefHeading___Toc401065150)

[Example ratings 11](#__RefHeading___Toc401065151)

[Domain 2: Identification and selection of studies 14](#__RefHeading___Toc401065152)

[Background 14](#__RefHeading___Toc401065153)

[Example ratings 16](#__RefHeading___Toc401065154)

[Domain 3: Data collection and study appraisal 18](#__RefHeading___Toc401065155)

[Background 18](#__RefHeading___Toc401065156)

[Example ratings 21](#__RefHeading___Toc401065157)

[Domain 4: Synthesis and findings 23](#__RefHeading___Toc401065158)

[Background 23](#__RefHeading___Toc401065159)

[Example ratings 28](#__RefHeading___Toc401065160)

[**Phase 3: Judging risk of bias** 30](#__RefHeading___Toc401065161)

[Example ratings 32](#__RefHeading___Toc401065162)

[**Appendix: Glossary** 38](#__RefHeading___Toc401065163)

# Tables

[Box: Examples of target questions and PICO equivalents for different types of systematic review 5](#__RefHeading___Toc420063469)

[Table 1: Signalling questions for Domain 1 with guidance on how to answer each question 9](#__RefHeading___Toc420063470)

[Table 2: Concerns regarding specification of study eligibility criteria 10](#__RefHeading___Toc420063471)

[Table 3: Example rating for specification of study eligibility criteria judged at high concerns 11](#__RefHeading___Toc420063472)

[Table 4: Example rating for specification of study eligibility criteria judged at low concerns 12](#__RefHeading___Toc420063473)

[Table 5: Signalling questions for Domain 2 with guidance on how to rate each question 15](#__RefHeading___Toc420063474)

[Table 6: Concerns regarding methods used to identify and/or select studies 16](#__RefHeading___Toc420063475)

[Table 7: Example rating for search strategy judged at high concerns 16](#__RefHeading___Toc420063476)

[Table 8: Example rating for search strategy judged at low concerns 17](#__RefHeading___Toc420063477)

[Table 9: Signalling questions for Domain 3 with guidance on how to rate each question 19](#__RefHeading___Toc420063478)

[Table 10: Concerns regarding methods used to collect data and appraise studies 20](#__RefHeading___Toc420063479)

[Table 11: Example rating for data collection and study appraisal judged at high concerns 21](#__RefHeading___Toc420063480)

[Table 12: Example rating for data collection and study appraisal judged at low concerns 22](#__RefHeading___Toc420063481)

[Table 13: Signalling questions for Domain 4 with guidance on how to rate each question 24](#__RefHeading___Toc420063482)

[Table 14: Concerns regarding methods used to synthesize results 27](#__RefHeading___Toc420063483)

[Table 15: Example rating for synthesis judged at high concerns 28](#__RefHeading___Toc420063484)

[Table 16: Example rating for synthesis judged at low concerns 29](#__RefHeading___Toc420063485)

[Table 17: Signalling questions for Phase 3 with guidance on how to rate each question 31](#__RefHeading___Toc420063486)

[Table 18: Risk of bias introduced by methods used to identify and/or select studies 31](#__RefHeading___Toc420063487)

[Table 19 Summary of concerns identified during the Phase 2 assessment 32](#__RefHeading___Toc420063488)

[Table 20: Example rating for synthesis judged at high risk of bias 33](#__RefHeading___Toc420063489)

[Table 21 Summary of concerns identified during the Phase 2 assessment 33](#__RefHeading___Toc420063490)

# The ROBIS Tool

The tool is completed in 3 phases: (1) assess relevance (optional), (2) identify concerns with the review process and (3) judge risk of bias in the review. Signalling questions are included to help assess specific concerns about potential biases with the review. The ratings from these signalling questions help assessors to judge overall risk of bias. In this document we take these three phases in turn, and provide guidance for the overall phase and for each signalling question. We refer the reader to more extensive resources offering guidance on conduct of systematic reviews for additional background. [^1^](#_ENREF_1) [^2^](#_ENREF_2) [^3^](#_ENREF_3) [^4^](#_ENREF_4)

## Definition of risk of bias

ROBIS assesses both the risk of bias in a review and (where appropriate) the relevance of a review to the research question at hand. Specifically, it addresses 1) the degree to which the review methods minimised the risk of bias in the summary estimates and review conclusions, and 2) the extent to which the research question addressed by the review matches the research question being addressed by its user (e.g. an overview author or guideline developer). *Bias* occurs if systematic flaws or limitations in the design, conduct or analysis of a review distort the results. Evidence from a review may have *limited relevance* if the review question did not match the overview/guidelines question.

## Target audience

ROBIS was developed with three specific user groups in mind:

- Authors of overviews of systematic reviews
- Guideline developers
- Reviewers who may want to assess risk of bias in their review once it is complete or to minimise the risk of bias when planning the review methods at the protocol stage

It may also be helpful for anyone who wants to assess the risk of bias in a systematic review, however, it has not been specifically designed for other target audiences. Other potential users of ROBIS include organisations supporting decision making (e.g. NICE, IQWiG), clinicians with an interest in evidence based medicine, journal editors and manuscript reviewers.

# Phase 1: Assessing Relevance (optional)

Assessors first report the question that they are trying to answer (e.g. in their overview or guideline) – we have called this the “target question”. For efficacy or effectiveness reviews they are asked to define this in terms of the PICO (participants, interventions, comparisons, outcomes). For reviews of different types of questions (e.g. diagnostic test, prognostic factors, aetiology or prediction models), alternative categories are provided as appropriate (see box). If a single review is addressing multiple question then it may be appropriate to complete a separate ROBIS assessment for each review question. Assessors complete the PICO or equivalent for the systematic review to be assessed using ROBIS, and are then asked whether the two questions (target question and systematic review question) match. If one or more of the categories (PICO or equivalent) do not match then this should be rated as “No”. If there is a partial match between categories then this should be rated as “partial”. For example, if the target question relates to adults, but the systematic review is restricted to participants aged more than 60 years. If a review is being assessed in isolation and there is no target question, then this phase of ROBIS can be omitted.

Box: Examples of target questions and PICO equivalents for different types of systematic review

| **Review Type** | **PICO equivalent** | **Example** |
| --- | --- | --- |
| Intervention[^5^](#_ENREF_5) | Patients/Population(s): | Adults with chronic hepatitis C virus infection |
|  | Intervention(s): | Triple anti-viral therapy with pegylated interferon |
|  | Comparator(s): | Dual anti-viral therapy |
|  | Outcome(s): | Sustained virologic response |
| Aetiology[^6^](#_ENREF_6) | Patients/Population(s): | Adults |
|  | Exposure(s) and comparator(s): | Body mass index |
|  | Outcome(s): | Colorectal cancer |
| Diagnosis[^7^](#_ENREF_7) | Patients): | Adults with symptoms suggestive of rectal cancer |
|  | Index test(s): | Endoscopic ultrasound |
|  | Reference standard: | Surgical histology |
|  | Target condition: | Early rectal cancer (T0) |
| Prognosis[^8^](#_ENREF_8) | Patients: | Pregnant women, with or without fetal growth restriction, no evidence of premature rupture of membranes, no evidence of congenital or structural anomalies. |
|  | Outcome to be predicted: | Adverse pregnancy outcome (low or high birth weight, neonatal death, perinatal mortality) |
|  | Intended use of model: | Prediction |
|  | Intended moment in time: | Late pregnancy (>37 weeks gestation) |

## Example Relevance Assessment[^7^](#_ENREF_7)

**For a diagnostic review:**

| **Category** | **Target question** | **Review being assessed**[**^7^**](#_ENREF_7) |
| --- | --- | --- |
| Patient(s): | Adults with symptoms suggestive of rectal cancer | Unclear |
| Index test(s): | Endoscopic ultrasound | Endoscopic ultrasound |
| Reference standard: | Surgical histology | Surgery |
| Target condition: | Early rectal cancer (T0) | Early rectal cancer (T0) |

| **Relevance assessment** |  | **Reasoning** |
| --- | --- | --- |
| Does the question addressed by the review match the question you are trying to answer (e.g. in your overview or guideline)? | Unclear | The question matches for the index test, reference standard and target condition categories but the population was not defined in the review and so it is unclear whether the question addressed by the review matches the target question. |

# Phase 2: Identifying concerns with the review process

Phase 2 aims to identify areas where bias may be introduced into the systematic review. It involves the assessment of four domains to cover key review processes: study eligibility criteria; identification and selection of studies; data collection and study appraisal; and synthesis and findings. This phase of ROBIS identifies areas of potential concern to help judge overall risk of bias in the final phase. Each domain comprises three sections: information used to support the judgment, signalling questions, and judgment of concern about risk of bias. The domains should be considered sequentially and not assessed as stand-alone units. For example, this means that, when assessing domain 2 (identification and selection of studies), the assessor should consider the terms and structure of the search strategy in relation to the research question specified in domain 1.

The signalling questions are answered as “Yes”, “Probably Yes”, “Probably No”, “No” and “No Information”, with “Yes” indicating low concerns. The subsequent level of concern about bias associated with each domain is then judged as “low,” “high,” or “unclear.” This rating was chosen rather than a rating of “Yes”, “No” or “Unclear”, because such a rating would either have meant that “Yes” was a bad thing in contrast to the phrasing of all signalling questions, or concern questions would have been worded awkwardly. Further, this rating was consistent with the rating of risk of bias in domain 3.If the answers to all signalling questions for a domain are “Yes” or “Probably Yes”, then level of concern can be judged as low. If any signalling question is answered “No” or “Probably No”, potential for concern about bias exists. The “No Information” category should be used only when insufficient data are reported to permit a judgment. By recording the information used to reach the judgment (*support for judgment*), we aim to make the rating transparent and, where necessary, facilitate discussion among review authors completing assessments independently. ROBIS users are likely to need both subject content and methodological expertise to complete an assessment.

## Phase 2, Domain 1: Study eligibility criteria

### Background

The first domain aims to assess whether primary study eligibility criteria were pre-specified, clear and appropriate to the review question. A systematic review should begin with a clearly focused question or objective.[^9^](#_ENREF_9) This should be reflected in the pre-specification of criteria used for deciding whether primary studies are eligible for inclusion in the review. This pre-specification aims to ensure that decisions about which studies to include are made consistently rather than on existing knowledge about the characteristics and findings of the studies themselves. It is usually only possible to assess whether eligibility criteria have been appropriately pre-specified (and adhered to in the review) if a protocol or registration document is available which pre-dates the conduct and reporting of the review. When no such document is available, assessors will need to base their judgement about this domain on the report of the review findings, making it difficult to know whether these criteria were actually stipulated in advance and governed what the reviewers did throughout the review, or whether they were decided or modified during the review process.

The eligibility criteria combine aspects of the review question with additional detail about the types of studies that the review will consider, including the study design. Ideally, the characteristics of eligible studies will be expressed according to the population/participants involved, the intervention/exposure evaluated and the comparisons made. The information provided about the characteristics of studies should be quite specific, as far as possible avoiding any ambiguities that might have allowed the reviewers to impose *post hoc* judgements about study eligibility during the review process. Reviewers will often impose restrictions in eligibility criteria, which may or may not be judged appropriate. For example, they might restrict their included studies by particular study characteristics, such as those reporting particular outcomes of interest, those meeting specific quality criteria, or those that are undertaken within a certain date range. They might also impose restrictions on the sources of information they will include, such as those that are published as full articles, or those that are available in English. Where any restriction is placed on the studies that are included, this must be stated in advance, and justification provided. In some circumstances, restrictions may introduce bias in the selection of studies that are eligible for inclusion.

Table 1 summarised the signalling questions included in Domain 1 together with rating guidance for each question. Table 2 provides an overview of domain level ratings for Domain 1. Example ratings of reviews where assessors/appraisers had high and low concerns for this domain are provided in Tables 3 and 4.

Table 1: Signalling questions for Domain 1 with guidance on how to answer each question

| **Signalling question** | **Rating guidance** |
| --- | --- |
| 1. Did the review adhere to pre-defined objectives and eligibility criteria? | A systematic review should begin with a clearly focused question or objective which is reflected in the criteria used for deciding whether studies are eligible for inclusion. Details that should be specified *a priori* in a review protocol vary according to review type, but should generally include the study designs, study participants, and types of interventions/exposures that are eligible. If outcomes or outcome domains are to form part of the eligibility criteria, this should be stated clearly. Any exclusions should also be pre-specified. Where a protocol providing this information is available, the answer to this question would be “Yes”. Where no protocol is available but information about pre-defined objectives and detailed eligibility criteria are supplied, and there is good reason to believe that these were specified in advance and adhered to throughout the review, assessors can consider answer this question “Probably Yes”. Any *post hoc* changes to the eligibility criteria or outcomes must keep faith with the objectives of the review, and be properly justified and documented. In the absence of a pre-published protocol, where information about pre-defined objectives and eligibility criteria are only available *post hoc* in the review publication, unless there is some reason to believe that these details were specified in advance and adhered to from the start of the review, this question should be answered “Probably No”. Where all or some of these details are missing, this question should be answered “No”. |
| 1. Were the eligibility criteria appropriate for the review question? | The eligibility criteria should stem from the review question and should provide sufficient detail to enable judgement about whether the studies that are included are appropriate to the question. The information required is likely to vary by topic. For example, in order to judge appropriateness, the assessor might need a clear description of the population in terms of the age range and diagnosis of the study participants, the setting in which the study was conducted, the dose of a drug, or the frequency of exposure. To answer this question the assessor is likely to require some content knowledge. |
| 1. Were eligibility criteria unambiguous? | Specific information about the characteristics of eligible studies must be provided, as far as possible avoiding any ambiguities about the types of study, population, interventions, comparators and outcomes. Criteria should be sufficiently detailed that the review could be replicated using the criteria specified. A number of important details are commonly missing from the eligibility criteria in systematic reviews. For example, details about the diagnosis of study participants. Diagnosis might be made using a number of different methods, some of which might be more valid or accurate than others. Review authors should have decided in advance which diagnostic methods are appropriate to their review question in order to avoid introducing potential biases during the review process. Similarly, specific details about interventions/exposures and comparators must be provided, including characteristics such as medication dose, frequency of administration, concurrent treatments, and so on. The assessor is likely to require some content knowledge to answer this question, but where specific queries remain about the stated eligibility criteria, “No” or “Probably No” judgements can usually be made. |
| 1. Were all restrictions in eligibility criteria based on study characteristics appropriate? | Any restrictions applied on the basis of study characteristics must be clearly described and a sound rationale provided. These details will enable assessors to judge whether such restrictions were appropriate. Examples might be the study design, the date the study was published, the size of the study, some measure of study quality, and available outcomes measures. This question is different from the one above which refers to whether the eligibility criteria are appropriate to the review question. Where sufficient information is available, and the assessor is reasonably satisfied that the restrictions are appropriate, this question can be answered “Yes or “Probably Yes”. Where restrictions around study characteristics are not justified and there is insufficient information to judge whether these restrictions are appropriate, this question should be answered “Probably No” or “No”. Where eligibility criteria are sufficiently detailed, and no restrictions around study characteristics are explicitly reported, it can be assumed that none were imposed, and the question should be answered “Yes”. |
| 1. Were any restrictions in eligibility criteria based on sources of information appropriate? | Any restrictions applied on the basis of sources of information must be clearly described and a sound rationale provided. These details will enable assessors to judge whether such restrictions were appropriate. Examples might be the publication status or format, language, and availability of data. This question is different from the question in domain 2 which is about restricting searches. Where eligibility criteria are sufficiently detailed, but no restrictions based on sources of information are explicitly reported, it must be assumed that none were imposed, and the question should be answered “Yes”. |

Table 2: Concerns regarding specification of study eligibility criteria

| Low concern | Considerable effort has been made to clearly specify the review question and objectives, and to pre-specify and justify appropriate and detailed eligibility criteria that have been adhered to during the review |
| --- | --- |
| High concern | Studies that would have been important and relevant to answering the review question are likely to have been excluded from the review, either due to the lack of pre-specified objectives and eligibility criteria, or because inappropriate restrictions were imposed or studies that are not appropriate for addressing the review question have been included. |
| Unclear concern | Insufficient information is reported to make a judgement about risk of bias. |

### Example ratings

#### a. Review judged at high concerns regarding specification of study eligibility criteria[^7^](#_ENREF_7)

Descriptions from the text:

Abstract – Aim

“To evaluate the accuracy of EUS in T staging of early rectal cancers.”

Study Selection Criteria

“Only EUS studies confirmed by surgical histology were selected. EUS criteria used for T0 was tumour confined to the mucosa. From this pool, only studies from which a 2 x 2 table could be constructed for true-positive, false-positive, false negative and true-negative values were included.”

****Table 3: Example rating for specification of study eligibility criteria judged at high concerns****

| **Signalling question** | **Rating** | **Reasoning** |
| --- | --- | --- |
| 1. Did the review adhere to pre-defined objectives and eligibility criteria? | No information | There was no evidence of pre-specification of objectives and eligibility criteria. |
| 1. Were the eligibility criteria appropriate for the review question? | Probably No | The eligibility criteria reported were appropriate, but some details were lacking in particular in relation to population. It was therefore not possible to judge whether eligibility criteria were appropriate. |
| 1. Were eligibility criteria unambiguous? | Probably No | There were insufficient details about eligibility criteria. In particular, there were no details about which study populations or which study designs are eligible. |
| 1. Were all restrictions in eligibility criteria based on study characteristics appropriate? | No information | No details about restrictions based on study characteristics were provided. Overall there is insufficient detail about eligibility criteria. |
| 1. Were any restrictions in eligibility criteria based on sources of information appropriate? | Probably Yes | The review was restricted to studies from which a 2x2 table could be constructed. Although no justification for this was provided this is common in DTA reviews and so is likely to have been reasonable. |
| Concerns regarding specification of study eligibility criteria | High | |
| Rationale for concern | There were insufficient details regarding study eligibility criteria to judge whether the appropriate studies were included in the review; in particular details on eligible participants were lacking. It was also unclear whether criteria were pre-specified or adapted post-hoc. | |

#### b. Review judged at low concerns regarding specification of study eligibility criteria[^8^](#_ENREF_8)

Descriptions from the text:

Objective

“To evaluate the association and predictive value of ultrasound measurements of amniotic fluid volume for adverse pregnancy outcome.”

Methods – Study selection

“1. Population. Pregnant women, with or without fetal growth restriction, no evidence of premature rupture of membranes, no evidence of congenital or structural abnormalities.

2. Index test. Any measure of amniotic fluid reported by the authors including AFI, amniotic fluid volume and maximum deepest pocket. Any threshold used to define low or high amniotic fluid as reported by the authors of the included studies was accepted.

3. Outcome. Any reference standard looking at compromise of fetal or neonatal wellbeing; including: abnormal cord pH at birth, Apgar scores, perinatal death and composite outcomes such as adverse perinatal outcome. Any reference standard for fetal growth restriction or small for gestational age: Birthweight <10^th^, <5^th^, <3^rd^ centile, absolute birthweight thresholds, ponderal index.

4. Study design. Observational studies in which the results of the test of interest are compared with the outcome findings as confirmed by a reference standard, allowing generation of a 2x2 table to compute indices of association and test accuracy for each available threshold. Case series of ten or fewer and case-control studies determined by outcome were excluded.”

Data Extraction

“All studies had to state that they excluded rupture of membranes and congenital/structural anomalies due to the association of renal/urinary tract anomalies and karyotypic anomalies with abnormalities of liquor volume.”

Table 4: Example rating for specification of study eligibility criteria judged at low concerns

| **Signalling question** | **Rating** | **Reasoning** |
| --- | --- | --- |
| 1. Did the review adhere to pre-defined objectives and eligibility criteria? | Yes | The authors specified clearly in the *Abstract* and *Background* of the article that the objectives were to evaluate the association and predictive value of ultrasound measurements of amniotic fluid volume for adverse pregnancy outcome. A separate protocol provided the specific review question “What is the accuracy of the amniotic fluid volume to predict fetal/neonatal compromise and fetal growth restriction?” The authors provided details of eligibility criteria in the *Study selection* section organised by population, index test, outcome and study design. |
| 1. Were the eligibility criteria appropriate for the review question? | Yes | The review question indicated the need to identify studies looking at the accuracy of the amniotic fluid volume to predict fetal/neonatal compromise and fetal growth restriction. The details of studies eligible for inclusion provided in the article appeared appropriate to the review question. |
| 1. Were eligibility criteria unambiguous? | Probably Yes | The types of study design were clearly stated. Observational studies were included. The authors excluded case series (of 10 or fewer) and case control studies determined by outcome. Clear details of the population of interest (pregnant women, with or without fetal growth restriction, no evidence of premature rupture of membranes, no evidence of congenital or structural anomalies), the index test (any reported measure of amniotic fluid regardless of threshold used, including AFI, amniotic fluid volume and maximum deepest pocket), outcome (any reference standard looking at compromise of fetal or neonatal wellbeing – several specific examples are listed; any reference standard for fetal growth restriction or small gestational age – again these are listed) were provided. |
| 1. Were all restrictions in eligibility criteria based on study characteristics appropriate? | Probably Yes | The restrictions based on types of study design and study size issues were clearly described. Further restrictions were provided in the Data Extraction section. The restrictions appeared to be appropriate, although no justification was provided. |
| 1. Were any restrictions in eligibility criteria based on sources of information appropriate? | Probably Yes | No language restrictions were applied. Some restrictions may have been based on the availability of data, although no justification is provided. No other restrictions on sources of information were described. Therefore this question was answered “Probably Yes”. |
| Concerns regarding specification of study eligibility criteria | Low |  |
| Rationale for concern | All signalling questions were answered as “Yes” or “Probably Yes”, so no potential concerns about the specification of eligibility criteria were identified. Considerable effort was made to clearly specify the review question and objectives, and to pre-specify and justify appropriate and detailed eligibility criteria. | |

## Phase 2, Domain 2: Identification and selection of studies

### Background

This domain aims to assess whether any primary studies that would have met the inclusion criteria were not included in the review. A sensitive search to retrieve as many eligible studies as possible is a key component of any systematic review. Ideally this search is carried out by or with guidance from a trained information specialist. Unbiased selection of studies based on the search results helps to ensure that all relevant studies identified by the searches are included in the review and that ineligible studies are not included. Searches should involve appropriate databases and electronic sources (which index journals, conferences and trial records) to identify published and unpublished reports, include methods additional to database searching to identify reports of eligible studies (e.g. checking references in existing reviews, citation searching, handsearching) and use of an appropriate and sensitive search strategy. Search strategies should include free-text terms (e.g. in the title and abstract) and any suitable subject indexing (e.g. MeSH or EMTREE) likely to identify relevant studies. It may also be helpful to search additional fields, such as CAS Registry Number, and drug or device trade name, depending on the topic and resource to be searched. Strategies are generally a combination of terms to capture one or more of the following concepts: population, intervention/index test/exposure. For certain types of review, for example reviews of RCTs, it may also be appropriate to include a methodological or study design filter (also known as hedges) in some or all of the databases searched. However, for other types of review such as those for diagnostic accuracy studies, the use of filters has been shown to miss relevant studies and so should be avoided. It can be difficult to assess the sensitivity of a search strategy without methodological knowledge relating to searching practice and content expertise relating to the review topic. In general, assessors should consider whether an appropriate range of terms is included to cover all possible ways in which the concepts used to capture the research question could be described. For example, for a review of breast cancer, just searching for the term “breast cancer” as a key word is not sufficient. An example of a strategy that would be expected for this population is shown below for the Medline database searched via the OvidSP interface:

1 exp breast neoplasms/ or Carcinoma, Ductal, Breast/ (214761)

2 exp breast/ (31443)

3 exp neoplasms/ (2533252)

4 2 and 3 (17379)

5 (breast$ adj5 (neoplasm$ or cancer$ or tumo?r$ or metasta$ or carcinoma$ or adenocarcinoma$ or sarcoma$ or dcis or ductal or infiltrat$ or intraductal$ or lobular or medullary)).ti,ab,ot,hw. (256928)

6 (mammar$ adj5 (neoplas$ or cancer$ or tumo?r$ or metasta$ or carcinoma$ or adenocarcinoma$ or sarcoma$ or dcis or ductal or infiltrat$ or intra-ductal$ or lobular or medullary)).ti,ab,ot,hw. (34861)

7 1 or 4 or 5 or 6 (277790)

Table 5 summarises the signalling questions included in Domain 2 together with rating guidance for each question. Table 6 provides an overview of domain level ratings for Domain 2. Example ratings of reviews where assessors hadhigh and low concerns for this domain are provided in Tables 7 and 8.

Table 5: Signalling questions for Domain 2 with guidance on how to rate each question

| **Signalling question** | **Rating guidance** |
| --- | --- |
| 1. Did the search include an appropriate range of databases/ electronic sources for published and unpublished reports? | The assessor needs to judge what constitutes an appropriate range of databases. This will vary according to review topic. It is anticipated that at a minimum a MEDLINE and EMBASE search would be conducted. Searches of material published as conference reports should also be considered along with a search of research registers. Guidance on the appropriate range of databases can be found in SR guidance such as the Cochrane Handbook,[^9^](#_ENREF_9) or from the Centre for Reviews and Dissemination (CRD) website (http://www.york.ac.uk/inst/crd/finding_studies_systematic_reviews.htm). |
| 1. Were methods additional to database searching used to identify relevant reports? | Additional methods such as citation searches, contacting experts, reference checking, handsearching etc. should have been performed. |
| 1. Were the terms and structure of the search strategy likely to retrieve as many eligible studies as possible? | A full search strategy showing all the search terms used, in sufficient detail to replicate the search, is required to be able to fully judge this question. If only limited details are provided, such as a list of search terms with no indication of how these are combined, assessors may be able to make a “Probably Yes” or “Probably No” judgment. Assessors should consider whether the search strategy included an appropriate range of terms for the topic, whether a combination of controlled terms (such as Medical Subject Headings (MeSH) for Medline) and words in the title and abstract were used, and whether any filters applied were appropriate. For example, for DTA reviews the use of filters has been shown to miss relevant studies and so this question should be answered as No for a strategy that includes such filters.[^10^](#_ENREF_10) Guidance on the critical appraisal of search strategies can be found in the PRESS Evidence-Based Checklist (<http://ejournals.library.ualberta.ca/index.php/EBLIP/article/view/7402>). |
| 1. Were restrictions based on date, publication format, or language appropriate? | If no restrictions were applied to the search strategy then this question should be answered as Yes. This is different from the question in domain 1 (1.5) which is about restriction to selection criteria. Information is required on all three components of this question (i.e. date, publication format and language) to be able to fully judge this item. Restriction of papers based on language (e.g. restriction to English language articles) or publication format (e.g. restriction to full text published studies) is rarely (if ever) appropriate, and so if any such restrictions were applied then this question should usually be answered as “No”. Restrictions on date may be appropriate but should be supported by a clearly described rationale for this question to be answered as “Yes”. For example, if a medication or test was not available before a certain date then it is reasonable to only start searches from the date at which the medication or test first became available. |
| 1. Were efforts made to minimise errors in selection of studies? | Both the process of screening titles and abstracts and of assessing full text studies for inclusion are covered by this question. Information on both are required to be able to fully judge this item. For an answer of “Yes”, titles and abstracts should be screened independently by at least two reviewers and full text inclusion assessment should involve at least two reviewers (either independently or with one performing the assessment and the second checking the decision). |

Table 6: Concerns regarding methods used to identify and/or select studies

| Low concern | Given the review question and eligibility criteria as assessed in Domain 1, a substantial effort has been made to identify as many relevant studies as possible through a variety of search methods using a sensitive and appropriate search strategy and steps were taken to minimise bias and errors when selecting studies for inclusion. |
| --- | --- |
| High concern | Some eligible studies are likely to be missing from the review. |
| Unclear concern | There is insufficient information reported to make a judgement on risk of bias. |

### Example ratings

#### a. Review judged at high concerns regarding methods used to identify and/or select studies [^5^](#_ENREF_5)

Description from the text:

“A research librarian searched Ovid MEDLINE from 1947 to August 2012, the Cochrane Library Database (through the first quarter of 2012), Embase (1976 to August 2012), Scopus (1960 to August 2012), PsychINFO (1806 to August 2012), clinical trials registries, and grants databases. At least 2 reviewers independently evaluated studies for inclusion. Non–English language articles were excluded. Included studies published as conference abstracts were only used in sensitivity analyses.”

****Table 7: Example rating for search strategy judged at high concerns****

| **Signalling question** | **Rating** | **Reasoning** |
| --- | --- | --- |
| 1. Did the search include an appropriate range of databases/ electronic sources for published and unpublished reports? | Yes | MEDLINE, EMBASE, Scopus, PsycINFO and the Cochrane library were searched (although it is not clear which resources within the Cochrane Library). This was judged to be an appropriate range. Clinical trials registries and grant databases were also searched. |
| 1. Were methods additional to database searching used to identify relevant reports? | No | No |
| 1. Were the terms and structure of the search strategy likely to retrieve as many eligible studies as possible? | No information | The full search strategy was not reported and there were no details of the search terms; there was therefore no information on which to base the assessment for this question. |
| 1. Were restrictions based on date, publication format, or language appropriate? | No | The review was restricted to English language studies; there is therefore a potential for publication bias. |
| 1. Were efforts made to minimise errors in selection of studies? | Probably Yes | Inclusion assessment is reported to have been conducted independently by at least two reviewers. However, it was not explicit that this applied to both screening search results and assessing full text articles. This item was therefore rated as “Probably Yes”. |
| Concerns regarding methods used to identify and/or select studies | High | |
| Rationale for concern | Restriction of the review to English language articles means that we think that there is a high risk that relevant studies have not been included in this review. We cannot judge whether the search strategy was fit for purpose. | |

#### b. Review judged at low concerns regarding methods used to identify and/or select studies [^8^](#_ENREF_8)

Description from the text:

“The following sources were searched from inception to October 2011: MEDLINE; EMBASE; Cumulative Index To Nursing And Allied Health Literature (CINAHL); The Cochrane Central Register of Systematic Reviews; The Cochrane Central Register of Controlled Trials; DARE; MEDION; SIGLE; Index of Scientific and Technical Proceedings, Web of Science and ClinicalTrials.gov database. The search consisted of keywords and MeSH terms relating to the tests under investigation combined with MeSH terms of ‘Prenatal Diagnosis’, ‘Ultrasonography’, ‘Amniotic Fluid’ and ‘Pregnancy Outcome’. The full search strategy is shown in the Appendix S2. The reference lists of all included primary and review articles were examined to identify cited articles not captured by electronic searches. No language restrictions were applied. The database was scrutinised by two reviewers (RKM, CHM) and full articles likely to meet the selection criteria were obtained. Translations were obtained for non-English articles. Three reviewers made the final inclusion/exclusion decisions according to adherence to the following criteria.”

Table 8: Example rating for search strategy judged at low concerns

| **Signalling question** | **Rating** | **Reasoning** |
| --- | --- | --- |
| 1. Did the search include an appropriate range of databases/ electronic sources for published and unpublished reports? | Yes | MEDLINE, EMBASE, CINAHL, SIGLE, Index of Scientific and Technical Proceedings, and ClinicalTrials.gov database, the Cochrane library, DARE and MEDION were searched. Web of Science was also searched although it was not clear which databases within this platform were searched. This was judged to be an appropriate range of resources and included attempts to locate published and unpublished reports. However The Cochrane Central Register of Systematic Reviews is likely to be a recording error and the authors searched CDSR. |
| 1. Were methods additional to database searching used to identify relevant reports? | Yes | The reference lists of all included primary and review articles were examined to identify cited articles not captured by electronic searches |
| 1. Were the terms and structure of the search strategy likely to retrieve as many eligible studies as possible? | Yes | A detailed search strategy was provided in a web appendix. This combined terms for the population (pregnant women) with terms for the intervention (ultrasonography) and appeared to be sensitive with no inappropriate restrictions (e.g. study design filter). |
| 1. Were restrictions based on date, publication format, or language appropriate? | Yes | Databases were searched from inception, no language restrictions were applied, and searches included steps to identify grey literature. The search was therefore judged not to have included any restrictions and so this item was answered as “Yes”. |
| 1. Were efforts made to minimise errors in selection of studies? | Probably Yes | The process for both screening titles and abstracts and assessment of full text papers was reported and included multiple reviewers. However, it was not explicit whether the reviewers acted independently and so this item was answered as “Probably Yes”. |
| Concerns regarding methods used to identify and/or select studies | Low | |
| Rationale for concern | All signalling questions were answered as “Yes” or “Probably Yes” and so no potential areas of bias were identified. The review is therefore likely to have included a high proportion of relevant studies. | |

## Phase 2, Domain 3: Data collection and study appraisal

### Background

The third domain aims to assess whether bias may have been introduced through the data collection or risk of bias assessment processes. Rigorous data collection should involve planning ahead at the protocol stage and using a structured data collection form that has been piloted. All data that will contribute to the synthesis and interpretation of results should be collected. These data should include both numerical and statistical data and more general primary study characteristics such as study design features, funding, setting, participant characteristics, selection criteria, intervention/exposure/index (diagnostic) test details, and participant withdrawals. The type of numerical data is dependent on the study designs included in the review. For example, for a DTA review 2x2 data of test performance are commonly extracted. For a review of observational studies, an adjusted effect estimate with an associated 95% confidence interval, details of variables adjusted for and method of analysis may be extracted. If data are not available in the appropriate format required to contribute to the synthesis, review authors should report how these data were obtained. For example, primary study authors may be contacted for additional data. Appropriate statistical transformations may be used to derive the required data. Data extraction creates the potential for error. Errors could arise from mistakes when transcribing data or failing to collect relevant information that is available in a study report. Bias may also arise from the process of data extraction which is, by its nature, subjective and open to interpretation. Duplicate data extraction (or single data extraction with rigorous checking) is therefore essential to safeguard against random errors and potential bias. [^2^](#_ENREF_2)

Validity of included studies should be assessed using appropriate criteria given the design of the primary studies included in the review. This assessment may be carried out using a validated tool developed specifically for studies of the design being evaluated, or may simply be a list of relevant criteria that may be important potential sources of bias. Whether a published tool or ad hoc criteria are used, the assessor should consider whether the criteria are sufficient to identify all important potential sources of bias in the included studies. The decision on whether criteria were sufficient should be based on empirical evidence regarding risk of bias in specific study designs. [^12-17^](#_ENREF_12) As with data extraction, bias or error can occur in the process of risk of bias assessment. Risk of bias assessment should, therefore, involve two reviewers, ideally working independently but at a minimum the second reviewer checking the decisions of the first reviewer. With both risk of bias assessment and data extraction, the process for resolving and discrepancies should be reported.

Table 9 summarises the signalling questions included in Domain 3 together with rating guidance for each question. Table 10 provides an overview of domain level ratings for Domain 3. Example ratings of reviews where assessors had high and low concerns for this domain are provided in Tables 11 and 12.

Table 9: Signalling questions for Domain 3 with guidance on how to rate each question

| **Signalling question** | **Rating guidance** |
| --- | --- |
| 1. Were efforts made to minimise error in data collection? | In order to minimize bias and errors in the data collection process this should involve at least two reviewers and structured data extraction forms that have gone through a piloting process should be used. Ideally this should be done independently but extraction by one reviewer and detailed checking by a second reviewer is also acceptable. Checking should involve the second reviewer reading the paper in detail to not only check the extracted data for accuracy but also to ensure that no relevant information was missed. |
| 1. Were sufficient study characteristics available for both review authors and readers to be able to interpret the results? | Information on study characteristics is essential to interpret the results of the review as this allows appropriate investigation of heterogeneity and consideration of the applicability of the results. This information may be reported in the methods section where information on the data collection is reported, it may be available from characteristics of included studies tables, or may be summarised in the text of the results. This question can be difficult to judge as all information collected as part of a review is not always presented in a publication, often due to space restrictions. Assessors may therefore need to access additional resources such as web appendices. |
| 1. Were all relevant study results collected for use in the synthesis? | Sufficient study results should be extracted to permit an appropriate synthesis to be carried out. Ideally, the review authors should report what data were required for the synthesis and in what format. For example, for a DTA review 2x2 data of test performance are commonly extracted. For a review of observational studies, an adjusted effect estimate with an associated 95% confidence interval, details of variables adjusted for and method of analysis may be extracted. If these data are not explicitly reported in the methods section it may be possible to work out what data were extracted from results tables, graphical summaries (e.g. forest plots) or data reported in the text. It may also be necessary to access additional resources such as web appendices or the review protocol if available.  It is very rare for all primary studies included in a review to report the data in the appropriate format required to contribute to the synthesis. For example, a review may require continuous data in the format of mean difference in change from baseline with associated 95% confidence interval. There are a variety of other measures which may be reported instead of this with studies often failing to report measures of variation. For a review to answer “Yes” to this question detailed information should be included in the methods section to describe how results data that were not reported in the format required for synthesis were obtained e.g. by estimating/transforming from reported data or by contacting authors for additional information. |
| 1. Was risk of bias (or methodological quality) formally assessed using appropriate criteria? | A formal risk of bias assessment is an essential component of any review. If risk of bias was not formally assessed then this question should be answered as “No”. If a formal assessment was carried out then, assessors will need to use their judgement regarding whether the criteria used were appropriate. If an accepted published tool was used for the appropriate design, such as the Cochrane Risk of Bias tool [^18^](#_ENREF_18) for RCTs or QUADAS-2 for DTA studies,[^19^](#_ENREF_19) then this will be fairly straightforward and this question can be answered as Yes. However, if the review simply lists the questions assessed, uses an unpublished tool, or a tool that is no longer recommended then this is more complicated. The assessor then needs to judge whether the criteria assessed were likely to identify potential sources of bias in the primary studies, given their study design. For example, the Jadad score has been very widely used but does not include allocation concealment which has been shown to be a major potential risk of bias in RCTs.[^20^](#_ENREF_20) To answer Yes to this question, reviews that have used the Jadad scale[^20^](#_ENREF_20) should therefore also have assessed allocation concealment. |
| 1. Were efforts made to minimise error in risk of bias assessment? | As with data collection, risk of bias assessment should involve at least two reviewers. Ideally this should be done independently but assessment by one reviewer and checking by a second reviewer is also acceptable. |

Table 10: Concerns regarding methods used to collect data and appraise studies

| Low concern | Given the studies included in the review as assessed in domain 2, risk of bias was assessed using appropriate criteria, data extraction and risk of bias assessment involved two reviewers, and relevant study characteristics and results were extracted. |
| --- | --- |
| High concern | Some bias may have been introduced through the data collection or risk of bias assessment processes. |
| Unclear concern | There is insufficient information reported to inform a judgement on risk of bias. |

### Example ratings

#### a. Review judged at high concerns regarding methods used to collect data and appraise studies [^7^](#_ENREF_7)

Description from the text:

“Tables of 2 x 2 were constructed with the data extracted from each study. Two authors independently searched and extracted the data into an abstraction form. Any differences were resolved by mutual agreement… There is no consensus on how to assess studies designed without a control arm. Hence, these criteria do not apply to studies without a control arm. Therefore, for this meta-analysis and systematic review, studies were selected based on completeness of data and inclusion criteria.”

**Comment:** The characteristics of included studies table only contains details on study design (retrospective or prospective), cancer type (all rectal cancer), and confirmatory procedure (surgery in all).

****Table 11: Example rating for data collection and study appraisal judged at high concerns****

| **Signalling question** | **Rating** | **Reasoning** |
| --- | --- | --- |
| 1. Were efforts made to minimise error in data collection? | Yes | Data extraction was performed independently by two reviewers using an abstraction form. Differences were resolved by agreement. |
| 1. Were sufficient study characteristics available for both review authors and readers to be able to interpret the results? | No | The summary table only included details on design (prospective or consecutive), type of cancer (all rectal) and type of confirmatory procedure (all surgery). No further details on study design or population were reported in the text. |
| 1. Were all relevant study results collected for use in the synthesis? | Yes | 2x2 data were extracted from each study which is sufficient to calculate all measures of diagnostic accuracy and associated confidence intervals and to perform a meta-analysis. |
| 1. Was risk of bias (or methodological quality) formally assessed using appropriate criteria? | No | Study quality was not formally assessed. The authors state that this is because there is no consensus on how to assess studies without a control arm but there are accepted tools for assessing DTA studies which could have been used. |
| 1. Were efforts made to minimise error in risk of bias assessment? | No | Study quality was not formally assessed. |
| Concerns regarding methods used to collect data and appraise studies | High |  |
| Rationale for concern | Lack of formal quality assessment means that the risk of bias in the included studies is unclear. There were insufficient study details available to allow the reader to interpret the results. There is therefore a high risk of bias in both the data collection and study appraisal process for this review. | |

#### b. Review judged at low concerns regarding methods used to collect data and appraise studies [^5^](#_ENREF_5)

Description from the text:

***“One investigator abstracted details about the study design, population, setting, interventions, analysis, follow-up, and results. A second investigator reviewed data for accuracy. Two investigators independently applied predefined criteria to assess study quality as good, fair, or poor. Discrepancies were resolved through consensus.”***

Table 12: Example rating for data collection and study appraisal judged at low concerns

| **Signalling question** | **Rating** | **Reasoning** |
| --- | --- | --- |
| 1. Were efforts made to minimise error in data collection? | Probably Yes | One reviewer performed the data collection and this was checked by a second reviewer for accuracy. It is unclear whether the second reviewer only checked whether the extracted data were accurate or also read the paper in detail to ensure that no relevant information was missed. This question was therefore answered as “Probably Yes” rather than “Yes”. |
| 1. Were sufficient study characteristics available for both review authors and readers to be able to interpret the results? | Yes | **Detailed study characteristics and results tables were provided in appendices which reported sufficient information for authors and readers to interpret results.** |
| 1. Were all relevant study results collected for use in the synthesis? | Yes | Dichotomous data were extracted as number of events and total number of patients in each treatment arm; these were used to calculate relative risks. This was appropriate for use in the synthesis. |
| 1. Was risk of bias (or methodological quality) formally assessed using appropriate criteria? | Probably Yes | **Details on the ROB criteria used were not provided but the authors cite the US Preventive Services Task Force methods guide (2001) which includes some appropriate criteria.** |
| 1. Were efforts made to minimise error in risk of bias assessment? | Yes | **Two investigators independently assessed study quality.** |
| Concerns regarding methods used to collect data and appraise studies | Low | |
| Rationale for concern | All signalling questions were rated as “Yes” or “Probably Yes” and so no potential areas of bias were identified. The review processes of data collection and study appraisal are therefore unlikely to have introduced bias into this review. | |

## Phase 2, Domain 4: Synthesis and findings

### Background

This domain aims to assess whether, given a decision has been made to combine data from the included primary studies (either in a quantitative or non-quantitative or synthesis), the reviewers have used appropriate methods to do so. Approaches to synthesis depend on the nature of the review question being addressed and on the nature of the primary studies being synthesized. For RCTs, a common approach is to take a weighted average of treatment effect estimates (on the logarithmic scale for ratio measures of treatment effect), weighting by the precisions of the estimates.[^21^](#_ENREF_21) Either fixed-effect or random-effects models can be assumed for this. However, there are many variants and extensions to this, with the options of modelling outcome data explicitly (for example, taking a logistic regression approach for binary data,[^22^](#_ENREF_22) of modelling two or more outcomes simultaneously (bivariate or multivariate meta-analysis,[^23^](#_ENREF_23) of modelling multiple treatment effects simultaneously (network meta-analysis[^24^](#_ENREF_24)), or of modelling variation in treatment effects (meta-regression[^25^](#_ENREF_25)), and these can be combined, making the synthesis very complex. Similar options are available for other types of review questions. For diagnostic test accuracy, a bivariate approach has become standard, in which sensitivity and specificity are modelled simultaneously to take account of their correlation.[^26^](#_ENREF_26) For some reviews, a statistical synthesis may not be appropriate and instead a non-quantitative or narrative overview of results should be reported.

Some of the most important aspects to consider in any synthesis (either quantitative or non-quantitative) are (i) whether the analytic approach is appropriate for the research question posed; (ii) whether between-study variation (heterogeneity) is taken into account; (iii) whether biases in the primary studies are taken into account; (iv) whether the information from the primary studies being synthesized is complete (particularly if there is a risk that missing data are systematically different from available data, for example due to publication or reporting bias); and (v) whether the reviewers have introduced bias in the way that they report their findings. Technical aspects of the meta-analysis method, such as the choice of estimation method, are unlikely to be an important consideration. However, mistakes may be important, such as interpreting standard errors as standard deviations, failing to adjust for design issues such as matched or clustered data, or applying the standard weighted average approach to risk ratios rather than their logarithms.

Table 13 summarises the signalling questions included in Domain 4 together with rating guidance for each question. Table 14 provides an overview of domain level ratings for Domain 4. Example ratings of reviews where assessors had high and low concerns for this domain are provided in Tables 15 and 16.

Table 13: Signalling questions for Domain 4 with guidance on how to rate each question

| **Signalling question** | **Rating guidance** |
| --- | --- |
| 1. Did the synthesis include all studies that it should? | The synthesis should generally seek to include all studies known to have collected data relevant to the question being addressed. Results from individual studies may be missing from the synthesis because the study is unknown to the reviewers (possibly due to publication bias): such missing studies are addressed under Domain 2 and by signaling question 4.5. This question addresses three further situations relating to the studies that have been identified for inclusion in the review: (i) the specific results from an included study are not available to the reviewers, (ii) the reviewers have failed to collect or process the data available, or (iii) the reviewers have purposefully excluded the results.  In case (i), it may be clear that included studies collected data but did not report the desired results. For example, a clinical trial might collect data for a clinical outcome of interest to the reviewers, but report no results for it. The important distinction here is again whether the result was likely to have been suppressed because of the finding (e.g. statistical significance) or simply not reported (e.g. for practical reasons such as paper length). If results are unavailable from one or more studies because the results were not statistically significant, this selective (non-)reporting will introduce bias into the synthesis of the studies that do contribute data.  In case (ii) [see also Domain 3, question 3.3], studies may have been omitted by mistake, or because the reviewers were unfamiliar with statistical computations that would allow their inclusion. This would be problematic if the omitted studies had systematically different results from those that were included.  As for case (iii), reviewers may make inappropriate decisions to exclude some studies from a synthesis. One potential example would be exclusion of studies deemed to be driving a large between-studies heterogeneity based on statistical considerations alone (although this may be reasonable as a sensitivity analysis).  To address these considerations, we recommend the assessor examines the numbers of included studies (e.g. from a flow chart) and the numbers of synthesized studies (e.g. in forest plots or tables). A mismatch would lead to consideration of why studies are not included in analyses. In reviews with large numbers of studies (e.g. more than 10 or 20 for a particular synthesis), funnel plots and related statistical analyses may provide hints as to the possibility of selective (non-)reporting bias within the studies: see question 4.5.[^16^](#_ENREF_16) |
| 1. Were all predefined analyses followed or departures explained? | The purpose of this question is to identify biases introduced by the reviewers through their selection of analyses and analysis methods in such a way that results they did not like are suppressed or replaced. In order to answer Yes the review should have followed a published or accessible protocol. Examples include registering a protocol with PROSPERO, an international prospective register of systematic reviews (http://www.crd.york.ac.uk/PROSPERO/) or publishing the protocol of a Cochrane review (http://www.thecochranelibrary.com/). If there is an indication that predefined analyses were followed, for example the methods section appears rigorous and all analyses mentioned are addressed in the results, then the assessor might answer “Probably Yes”. In the explicit absence of a pre-specified protocol, the assessor should answer this question as “No”. However, if the systematic review makes no reference to the existence or absence of a protocol we recommend that the assessor answer this question as “no information”. |
| 1. Was the synthesis appropriate given the nature and similarity in the research questions, study designs and outcomes across included studies? | This question primarily addresses the methodology of the synthesis. The methodology should be driven by the nature of the studies and the nature of the question being asked, so it is important to consider these aspects as part of the assessment of the synthesis methodology.  If a quantitative synthesis is undertaken (a meta-analysis), this question both addresses whether it was appropriate to do this and addresses the statistical methods used. If a narrative approach is used for the synthesis, this question addresses both whether a quantitative synthesis would have been more appropriate and whether the particular narrative approach was appropriate.  The decision as to whether a quantitative synthesis is appropriate is driven primarily by the similarity of the studies in their research questions, designs and outcomes (where measured). This is often referred to as ‘clinical heterogeneity’ or ‘clinical diversity’. It is important that the assessor can be convinced that the result of the synthesis has meaning – i.e. can potentially inform policy, practice or further research. The issue of *statistical* heterogeneity is explicitly addressed in the following question.  The assessor should answer this question “Yes” if an ‘accepted’ method of statistically combining studies is used, including the appropriate weighting of each study, was used. For example in the presence of between-study variation, but where treatment effects can be assumed to come from a common distribution, a random effects meta-analysis model might be considered appropriate but a fixed effect model might not. But note that simple random-effects meta-analyses are not appropriate when there is a strong relationship between study size and effect size (‘small study effects’ or ‘funnel plot asymmetry’). Regression approaches or subgrouping might be considered when there are sufficient studies to explore reasons for between-study variation. We do not generally recommend methods of combining studies that ignore the influence of study precision on the overall pooled estimate – i.e. those that weight all studies equally. If such methods are used (e.g. vote counting) we suggest the assessor answers “No” to this question.  For studies of diagnostic test accuracy, a commonly-accepted method is a bivariate approach to account for the correlation between sensitivity and specificity. Statistical advice might be required for meta-analyses using multivariate, multilevel, Bayesian or network meta-analysis approaches. |
| 1. Was between-studies variation (heterogeneity) minimal or addressed in the synthesis? | This question targets variation in results of the studies rather than the variation in their characteristics. Between-study variation might be assessed visually (e.g. forest plot); using a statistical test (e.g. Cochran’s χ^2^ test, or Q); using a measure of between-study variance (often referred to as τ^2^); or using a measure of confidence interval overlap (e.g. the I^2^ inconsistency statistic).  If substantial heterogeneity is ignored in a meta-analysis, it can lead to misleading conclusions and/ or to spurious precision. If a fixed-effect (or fixed-effects) analysis is used in the presence of heterogeneity, it is important that the reviewers acknowledge that the analysis ignores the heterogeneity. The result should be supplemented with results regarding between-study variation, and the results from the fixed-effect(s) analysis should be interpreted appropriately. In most cases, the use of a fixed-effect(s) meta-analysis without further analyses to explore the source of the variation would receive a “No” answer for this question.  If a random-effects model has been used appropriately to allow for heterogeneity and/or further subgroup/ meta-regression analyses run to explore heterogeneity, the assessor might answer “Yes”. However, random-effects meta-analysis of studies that are extremely diverse in either characteristics or results may yield a meaningless result, particularly if the results of the studies point in opposing directions of effect.  If a narrative synthesis was conducted on the basis that a statistical combination was inappropriate due to clinical heterogeneity this question should be answered “Yes”, since heterogeneity was addressed by not combining. |
| 1. Were the findings robust, e.g. as demonstrated through funnel plot or sensitivity analyses? | This question addresses the precariousness of the findings from the synthesis, and whether they could change by altering the approach to synthesis. Funnel plots are used to examine relationships between effect size and study size (often measured statistically using precision). Asymmetry in a forest plot could be due to several reasons including publication bias, within-study (non-)reporting bias, different magnitudes of bias in larger vs smaller studies, or genuine differences in effects underlying studies of different sizes. When a funnel plot is asymmetrical, standard fixed-effect and random-effects methods produce different results. It is often therefore informative to undertake both methods as a form of sensitivity analysis. Alternatively, the trim-and-fill method, regression approaches or use of selection models may be used to examine the potential impact of different types of publication bias on the findings.[^27^](#_ENREF_27)  Other examples of sensitivity analyses are the use of different methods to impute missing data, or the exclusion of single studies thought to strongly drive the result of the synthesis. Alternatively, a sensitivity analysis may be performed to check whether findings are robust to removal of studies that did not use formal diagnostic criteria for disease, or studies that were rated at high risk of bias.  The use of sensitivity analysis per se is not considered adequate to receive an answer of “Yes” to this question; assessors should decide whether their use demonstrated robustness of findings. If there are very few studies or very heterogeneous studies, it may be clear that the findings are not robust even if the reviewers did not implement sensitivity analyses.  If a narrative synthesis was conducted, the assessor should consider whether different approaches to summarizing the studies could have led to different conclusions, and whether single studies are driving the conclusions. |
| 1. Were biases in primary studies minimal or addressed in the synthesis? | Assessors are encouraged to answer this question as “No” if they judge there to be important bias in constituent studies that has been ignored by the reviewers. For example, if risk of bias has not been evaluated in the systematic review ROBIS assessors should answer “No”. Equally, if risk of bias has been assessed but reviewers have not incorporated it into findings/ conclusions this should also receive a “No” answer to this question. Examples of when this question would be answered “Yes” might include all studies having received a “low risk of bias” rating from the reviewer or sensitivity analyses/ adjustment approaches were employed where studies were at high risk of bias. If biases are addressed only as part of the discussion of the findings from a synthesis, the assessor should answer “No”; such discussions are addressed later in the overall judgement on risk of bias in the review (Phase 3). |

Table 14: Concerns regarding methods used to synthesize results

| Low concern | The synthesis is unlikely to produce biased results, because any limitations in the data were overcome, or the findings were so convincing that the limitations would have little impact. |
| --- | --- |
| High concern | The synthesis is likely to produce biased results, because (i) potential biases were ignored (within and/or across studies), (ii) important between-study variation was not accounted for; (iii) there were important inadequacies in the methodology; or (iv) findings are incompletely reported in a way that raises concerns. |
| Unclear concern | There is insufficient information reported to make a judgement on risk of bias. |

### Example ratings

#### a. Review judged at high concerns regarding the synthesis[^28^](#_ENREF_28)

****Table 15: Example rating for synthesis judged at high concerns****

| **Signalling question** | **Rating** | **Reasoning** |
| --- | --- | --- |
| 1. Did the synthesis include all studies that it should? | No information | The author stated that 35 studies were relevant to the review, of which 29 were useable in a meta-analysis. No other information was provided to verify this. |
| 1. Were all predefined analyses followed or departures explained? | No information | No analyses were predefined in an explicitly referenced protocol. No further information was given in the text. |
| 1. Was the synthesis appropriate given the nature and similarity in the research questions, study designs and outcomes across included studies? | No | The author stated only that a “meta-analysis was performed” (p.11). The author summed the events and populations and used the totals to generate the summary odds ratio, so has not exploited within-study comparisons. The author described the studies to be pooled, but it would appear there is clinical diversity across the studies. |
| 1. Was between-studies variation (heterogeneity) minimal or addressed in the synthesis? | Probably no | No detail was provided on statistical heterogeneity. However, the author did discuss subgroup analyses for high risk, partner study and random population studies. Also, studies were grouped according to prevalence of circumcision in the community. No further detail was given on the subgroups. |
| 1. Was robustness of the finding(s) assessed e.g. through funnel plot or sensitivity analyses? | Probably no | The author discussed some studies that may be problematic. “Although these may be erroneous assumptions, the inclusion or exclusion of these studies did not affect the overall outcome” (P.11). Odds ratios and confidence intervals for these analyses were not reported. |
| 1. Were biases in primary studies minimal or addressed in the synthesis? | No | The studies were not explicitly evaluated for quality or risk of bias. Bias was not explicitly addressed in the synthesis. |
| Concerns regarding the synthesis and findings | High | |
| Rationale for concern | Individual studies were not assessed for risk of bias, nor was potential bias accounted for in the synthesis. The author summed intervention groups across studies rather than analyzing within-study comparisons, which is a dangerous approach to meta-analysis. There was no discussion or assessment of heterogeneity in the analysis. | |

#### b. Review judged at low concerns regarding the synthesis [^8^](#_ENREF_8)

Table 16: Example rating for synthesis judged at low concerns

| **Signalling question** | **Rating** | **Reasoning** |
| --- | --- | --- |
| 1. Did the synthesis include all studies that it should? | No information | Flow chart reported 43 studies included in the review. A separate figure reporting QUADAS-2 ratings also showed 43 studies. An appendix with details of study characteristics was included which also reported 43 studies. However, they reported summary forest plots which do not present findings by study, as studies may report more than one outcome. It is not possible to verify whether all studies were included in these syntheses. |
| 1. Were all predefined analyses followed or departures explained? | Probably yes | A protocol was available as supplementary information. No departures were stated or observed. Although the protocol did not specify detail on risk-of -bias assessment or strategy for analysis, the methods section of the article addresses each in a rigorous manner. All analyses in methods section are addressed in results. |
| 1. Was the synthesis appropriate given the nature and similarity in the research questions, study designs and outcomes across included studies? | Yes | The authors state “results were pooled using a random effects meta-analysis model where the definition of the measure of amniotic fluid volume, the threshold used and the outcome measure were the same” (page 688). To assess the predictive ability of the tests authors calculated summary sensitivity, specificity and likelihood ratios, using data from 2 × 2 tables and synthesised using a bivariate random-effects meta-analysis model. |
| 1. Was between-studies variation (heterogeneity) minimal or addressed in the synthesis? | Yes | Statistical heterogeneity was anticipated in this review and addressed using a random effects model. Meta-regression or subgroup analyses were performed to examine the effect of potential effect modifiers. Prediction intervals were also used when *I^2^* >0%. |
| 1. Was robustness of the finding(s) assessed e.g. through funnel plot or sensitivity analyses? | Probably no | Authors did not state whether sensitivity analyses were used to assess the robustness of their findings. However, there is discussion around the use of a composite outcome. |
| 1. Were biases in primary studies minimal or addressed in the synthesis? | Probably yes | Biases were assessed using the QUADAS-2 tool. This assessment indicated that adequate description of the reference standard and blinding for the reference standard were at high risk of bias. Other aspects of QUADAS-2 were not of major concern. “Quality” was stated as a subgroup analysis but subgroup analyses were only reported for one of the two primary outcomes. For oligohydramnios & fetal well-being outcome the authors stated that “There was no difference in any of the subgroup analyses” (P.691). |
| Concerns regarding the synthesis and findings | Low | |
| Rationale for concern | Authors satisfactorily addressed heterogeneity in their analysis and explored using subgroup analyses. Risk of bias of the individual studies was addressed and authors stated it was included as a subgroup analysis. | |

# Phase 3: Judging risk of bias

The final phase considers whether the systematic review as a whole is at risk of bias. This assessment uses the same structure as the phase 2 domains, including signalling questions and information used to support the judgement, but the judgement regarding concerns about bias is replaced with an overall judgement of risk of bias. The first signalling question for this phase asks whether the interpretation of findings addresses all of the concerns identified in domains 1 to 4. If no concerns were identified then this can be answered as “Yes”. If one or more concerns were identified for any of the previous domains, but these were appropriately considered when interpreting results and drawing conclusions, then this may also be rated as “Yes” and, depending on the rating of the other signalling questions, the review may still be rated as “Low risk of bias”.

This phase also includes a further three signalling questions relating to the interpretation of the review findings. These include whether the review conclusions were supported by the evidence presented, whether the relevance of the included studies was considered, and whether reviewers avoided emphasising results on the basis of their statistical significance. These are all important aspects of the interpretation of the review findings where bias may be introduced into the review.

Table 17 summarises the signalling questions included in Domain 4 together with rating guidance for each question. Table 18 provides an overview of domain level ratings for Domain 4.

Table 17: Signalling questions for Phase 3 with guidance on how to rate each question

| **Signalling question** | **Rating guidance** |
| --- | --- |
| 1. Did the interpretation of findings address all of the concerns identified the Phase 2 assessment? | This question refers back to the assessment of concerns regarding the review process performed in Phase 2 and summarized at the start of Phase 3. If all the domains assessed during phase 2 were rated as “Low concern” then this question can be answered as “Yes”. If one more was rated as “High concern” or “Unclear concern” then reviewers should consider whether the review authors have appropriately addressed concerns identified during phase 2 in the interpretation of findings. For example, consider a review that was judged at high risk of bias because it applied a study design filter to the searches meaning that relevant studies may have been missed. If this review acknowledged this as a potential limitation and discussed how this could have impacted on the findings of the review in the discussion and conclusions of the review then this signaling question can be rated as “Yes”. |
| 1. Was the relevance of identified studies to the review's research question appropriately considered? | An important aspect when interpreting the review findings is to consider the relevance (applicability/external validity) of the identified (included) studies to the review’s research question. If the studies included in the review are not directly applicable to the studies research question and this is not considered when interpreting the review findings then bias may be introduced. Some reviews may consider the relevance of the included studies as part of the formal quality assessment. This is particularly likely for diagnostic accuracy reviews that have used the QUADAS-2 tool [^29^](#_ENREF_29) which includes formal assessment of the applicability of the primary studies to the review question, as well as assessing risk of bias in the primary study. Other reviews may use a less formal approach, with a discussion of the relevance of the studies in the discussion section of the review. Either approach can be appropriate as long as the conclusions of the review present an appropriately reflection of the evidence including the relevance of the included studies. |
| 1. Did the reviewers avoid emphasizing results on the basis of their statistical significance? | If multiple analyses are reported in a single review it is important that reviewers present a balanced account of all analyses. Sometimes review authors may choose to highlight results on the basis of their statistical significance. For example, if a review evaluated 10 outcomes and found no effect for 9 outcomes but a significant beneficial effect for 1 then it can be tempting to overemphasize that outcome. However, this would give a misleading picture of the true results of the review findings and so it is important that this should not be done. |

Table 18: Risk of bias introduced by methods used to identify and/or select studies

| Low risk of bias | The findings of the review are likely to be reliable. Phase 2 did not raise any concerns with the review process or concerns were appropriately considered in the review conclusions. The conclusions were supported by the evidence and included consideration of the relevance of included studies. |
| --- | --- |
| High risk of bias | One or more of the concerns raised during the Phase 2 assessment was not addressed in the review conclusions, the review conclusions were not supported by the evidence, or the conclusions did not consider the relevance of the included studies to the review question. |
| Unclear risk of bias | There is insufficient information reported to make a judgement on risk of bias. |

### Example ratings

#### a. Review judged at high risk of bias[^7^](#_ENREF_7)

Table 19 Summary of concerns identified during the Phase 2 assessment

| **Domain** | **Concern** | **Rationale for concern** |
| --- | --- | --- |
| 1. Concerns regarding specification of study eligibility criteria | High | Eligibility criteria were not clear and unambiguous; details on eligible populations were not reported. |
| 2. Concerns regarding methods used to identify and/or select studies | High | It is likely that relevant studies were missed by the searches. Although limited details were reported it does not appear that an appropriate range of databases were searched, the search strategy included diagnostic study design terms, methods additional to database searching were not reported, and the restriction of the searches to 1980 onwards was not justified. |
| 3. Concerns regarding used to collect data and appraise studies | High | Lack of formal quality assessment means that the risk of bias in the included studies is unclear. There were insufficient study details available to allow the reader to interpret the results. There is therefore a high risk of bias in both the data collection and study appraisal process. |
| 4. Concerns regarding the synthesis | High | Although the methods of analysis used in this review were not the most statistically robust overall they appear likely to be reliable. The main concern with this review is that heterogeneity, and in particular the one outlying result was not investigated. As very limited study details are presented and a formal risk of bias assessment was not conducted the reader cannot assess whether this study is different from the other studies. If this study is likely to be more reliable than the other studies included in the review then the overall findings of the review will be biased. |

Description from the text:

“*This meta-analysis and systematic review shows that the pooled sensitivity and specificity of EUS for T0 tumor invasion is very high (around 97%)… Heterogeneity among different studies was evaluated not only with test of heterogeneity but also by drawing SROC curves and finding the AUC, since different studies might use slightly different criteria for staging. An AUC of 1 for any diagnostic test indicates that the test is excellent. SROC curves for EUS showed that the value of AUC was very close to 1, indicating that EUS is an excellent diagnostic test for T0 stage of rectal cancers….EUS has excellent sensitivity and specificity, which helps to accurately diagnose T0 stage of rectal cancers..*”

****Table 20: Example rating for synthesis**** judged at high risk of bias

| **Signalling question** | **Rating** | **Reasoning** |
| --- | --- | --- |
| 1. Did the interpretation of findings address all of the concerns identified the Phase 2 assessment? | No | None of the limitations identified by the Phase 2 assessment were identified as limitations by the review authors and so were not addressed in the interpretation of findings. |
| 1. Was the relevance of identified studies to the review's research question appropriately considered? | No | The review did not consider the relevance of the included studies to the review question and there were insufficient details, especially in relation to population, for the reader to make this assessment. |
| 1. Did the reviewers avoid emphasizing results on the basis of their statistical significance? | Yes | The figures emphasized in the discussion and on which the conclusions are based are all summary estimates from the primary analysis. |
| Risk of bias | High | |
| Rationale for concern | The phase 2 assessment identified a number of areas of concern with the review process which were not addressed by the authors. These include lack of clarity in inclusion criteria, possibility of missing studies, lack of formal quality assessment, insufficient details on included studies, and failure to appropriately consider differences between studies in the synthesis. The review also does not consider the relevance of the identified studies to the review’s research question. There is therefore a high risk of bias in this review. | |

#### b. Review judged at low risk of bias

Table 21 Summary of concerns identified during the Phase 2 assessment

| **Domain** | **Concern** | **Rationale for concern** |
| --- | --- | --- |
| 1. Concerns regarding specification of study eligibility criteria | Low | All signalling questions were answered as “Yes” or “Probably Yes”, so no potential concerns about the specification of eligibility criteria were identified. Considerable effort was made to clearly specify the review question and objectives, and to pre-specify and justify appropriate and detailed eligibility criteria. |
| 2. Concerns regarding methods used to identify and/or select studies | Low | The process for both screening titles and abstracts and assessment of full text papers was reported and included multiple reviewers. However, it was not explicit whether the reviewers acted independently and so this item was rated as “Probably Yes. |
| 3. Concerns regarding used to collect data and appraise studies | Low | All articles were assessed independently by a minimum of two reviewers and appropriate data were abstracted independently. Study quality was formally assessed using an appropriate tool. |
| 4. Concerns regarding the synthesis and findings | Low | Authors satisfactorily addressed heterogeneity in their analysis and explored using subgroup analyses. Risk of bias of the individual studies was addressed and authors stated it was included as a subgroup analysis. |

Description from the text:

Conclusions

“Current evidence suggests that oligohydramnios is strongly associated with being small for

gestational age and mortality, and polyhydramnios with birthweight >90th centile. Despite strong associations with poor outcome, they do not accurately predict outcome risk for individuals”

Discussion

“Our quality assessment revealed concerns regarding possibility of bias through patient selection, performance of the index test and reference standard. We were unable to perform subgroup analysis

for preterm versus term pregnancies and some studies reported insufficient data to determine whether thresholds for amniotic fluid measurement were adjusted for gestation. Where possible we used the results obtained closest to delivery and have performed subgroup analysis for those where the test was performed within 7 days of delivery. In particular, there was very poor reporting regarding the exact methods of the reference standards and whether there was any treatment used between the performance of the index and reference standard. A major concern therefore is in how many pregnancies was induction of labour performed due to the finding of oligohydramnios, which influences the results for pregnancy outcome, i.e. intervention bias. This bias can only truly be removed by performing an RCT, this would be impossible to perform as measurements of amniotic fluid volume have become the standard in fetal surveillance and management of high-risk pregnancies and so recruitment to such a trial would be very difficult. Finally, the outcome measures used in this review were those that were reported by the authors of the included studies, it is recognised that many of the outcome measures are subjective (e.g. admission to neonatal intensive care unit, need for resuscitation). The only real objective measure of poor fetal outcome is paired samples of cord pH and longer-term outcomes such as cerebral palsy, which were not reported.”

**Table 22: Example rating for synthesis judged at low** risk of bias

| Signalling question | Rating | Reasoning |
| --- | --- | --- |
| 1. Did the interpretation of findings address all of the concerns identified during the Phase 2 assessment? | Yes | There were no concerns identified during the phase 2 assessment. |
| 1. Was the relevance of identified studies to the review's research question appropriately considered? | Probably Yes | Not explicitly but the implications of the review findings at the individual level was discussed in detail. The potential sources of bias in terms of the populations and outcomes available from the included studies were discussed in detail which also have implications for relevance. |
| 1. Did the reviewers avoid emphasizing results on the basis of their statistical significance? | Yes | The review conclusions reflect both the statistically significant and non-significant review findings. |
| Risk of bias | Low | |
| Rationale for risk | The phase 2 assessment identified no concerns with the review process. The potential limitations of the studies included in the review in terms of risk of bias were discussed in detail in the discussion. The review conclusions appropriately reflect the results of the review. | |

**Practical issues when using ROBIS**

Judging risk of bias in systematic reviews is not always straightforward. ROBIS is designed to provide a structured approach to evaluate risk of bias, with an optional assessment of applicability. While ROBIS has been designed to be usable by reviewers with different backgrounds, some methodological and content expertise is likely to be required. It is a recommended that a ROBIS assessment is completed by two reviewers, ideally independently but at a minimum one reviewer with a second reviewer checking the assessment. We recommend that all signaling questions are considered for a ROBIS assessment. ROBIS has been developed to be generic to apply to reviews covering a variety of topics and as such all signaling questions should be relevant to all review types.

ROBIS involves making judgements. We have aimed to make this process as transparent as possible by asking reviewers to record the information used to support the judgements, the signalling questions and recording the rationale for the overall concern judgements.

A full ROBIS assessment requires that all signaling questions for all domains are assessed. However, it may be appropriate in certain situations to “stop” once a certain rating is achieved. This is dependent on the purpose of the assessment. If the aim of the ROBIS assessment is to provide an assessment of the risk of bias in the review and reasons for any bias then a full assessment will be required. However, if the purpose is to simply identify whether the review is at high risk of bias overall or whether there are concerns with particular domains then assessors may choose to stop once a high concern/risk of bias has been identified. For example, if a review did not search an appropriate range of databases (signaling question 2.1) then the review may be considered at high concerns for this domain. Assessors only interested in whether the review is at risk of bias may then choose not to assess the rest of the signaling questions in this domain or any of the other domains, simply assigning a rating of “high” risk of bias.

**References**

# Appendix: Glossary

This section provides an overview of some of the terms used in ROBIS with definitions relating to how these terms are used within ROBIS.

| **Term** | **Definition** |
| --- | --- |
| Bias | Systematic flaws or limitations in the design, conduct or analysis of a review (or primary study) that distort the results |
| Data collection | The process of collecting data from primary study reports and other sources; sometimes referred to as data extraction. May involve collecting information from additional sources (e.g. contacting authors) as well as simply extracting data from primary study reports. |
| Databases | Bibliographic databases that index study reports |
| Electronic sources | Online sources (other than bibliographic databases) used to locate reports of studies for inclusion in systematic reviews. |
| Eligibility criteria | The criteria used to determine whether a study should be included or excluded from a systematic review. Also known inclusion/exclusion criteria. |
| Guideline | Systematically developed statements to assist practitioners and patients to make decisions about appropriate health care for specific clinical circumstances. They should be based on the best available evidence. This means that guideline developers generally have to include and appraise existing systematic reviews in their guidance. |
| Heterogeneity | Variability across studies. Heterogeneity can be used in the general sense to describe differences between studies in terms of population, intervention, comparator, methodological quality or study design. Statistical heterogeneity occurs when effect estimates between studies differ to a greater extent than would be expected because of sampling variation (chance). |
| Meta-analysis | The statistical method for combining the results of a number of studies. This is done by calculating a weighted average of the effect estimates from different studies. A meta-analysis is often conducted as part of a systematic review but is not always possible or appropriate. |
| Overview | A review of systematic reviews. |
| Overview authors | The authors of the overview. |
| Readers | The person reading the systematic review |
| Relevance | The extent to which the review question matches the question that you are interested in. |
| Reports | A summary of the studies methods, findings and conclusions, these may be published or unpublished. A single study may be reported in multiple different reports, some single reports may report multiple studies. |
| Review | Short term for a systematic review. Can also be used to mean a non-systematic review article but this is not its meaning in the context of ROBIS. |
| Review authors | The authors of the systematic review; sometimes simply referred to as “reviewers”. |
| Search strategy | The exact terms and their combinations used to search bibliographic databases. |
| Statistical significance | The probability that the observed result or one that is more extreme, given that a certain statement (the null hypothesis) is true is caused by chance. Statistical hypothesis testing is used to determine whether the results of a study can lead to the rejection of the null hypothesis. A threshold for rejection of the null hypothesis of 5% is often set, but any other value can be chosen. Thus, if a *p*-value is found to be less than 0.05 (or the specified level), then the result would be considered statistically significant and the null hypothesis would be rejected. |
| Studies | The primary studies include in a systematic review. A single study may be reported in multiple reports. |
| Summary estimate | A single estimate of effect derived from multiple studies using meta-analysis. |
| Synthesis | The analysis, both quantitative and non-quantitative, used to summarise the results of the primary studies included in the systematic review. |
| Systematic review | A systematic approach to reviewing and summarising evidence from studies. They follow a defined structure to identify, evaluate and summarise all available evidence addressing a particular research question. They may include a meta-analysis, but this is not requirement of a systematic review. |
| Target question | The question that assessors are trying to answer for example in their overview or guideline. |
